# Supplementary material for: Bacterial N4-methylcytosine as an epigenetic mark in eukaryotic DNA
Source: Nat Commun. 2022 Feb 28;13:1072. doi: 10.1038/s41467-022-28471-w (PMC8885841; doi:10.1038/s41467-022-28471-w)
Supplement: Supplementary file 1 — Supplementary Information [file 41467_2022_28471_MOESM1_ESM.pdf]

Supplementary Information

Bacterial N4-methylcytosine as an epigenetic mark in eukaryotic DNA

Fernando Rodriguez[1], Irina A. Yushenova[1], Daniel DiCorpo[1,2], Irina R. Arkhipova[1]*

[1]Josephine Bay Paul Center for Comparative Molecular Biology and Evolution, Marine Biological Laboratory, Woods Hole, MA 02543, USA

[2]Present address: Department of Biostatistics, Boston University School of Public Health, Boston, MA 02118, USA

*Correspondence to Irina R. Arkhipova: iarkhipova@mbl.edu

Table of Contents

Supplementary Figures **2**

Supplementary Figures 1-17 2

Supplementary Tables **26**

Supplementary Table 1. Comparison of N6A methylation in *A. vaga* and other eukaryotes 26

Supplementary Table 2. Putative amino-MTase and demethylase orthologs in the phylum Rotifera 27

Supplementary Table 3. Properties of *E. coli* strains 27

Supplementary Table 4. Genometric correlations between DIP-seq methylation marks and gene and TE annotations in Av-ref and AvL1 assemblies 28

Supplementary Table 5. Genome assembly and gene annotation metrics 29

Supplementary Table 6. SMRT-seq base modification detection 30

Supplementary Table 7. Primers and oligonucleotides 31

Supplementary Table 8. N4CMT recombinant proteins 32

Supplementary Table 9. Summary of N4CMT action on *E. coli* genomic DNA in vitro 32

Supplementary Table 10. Summary of ChIP-seq peaks identified by MACS2 and overlap of peaks within Av-ref and AvL1 assemblies 32

Supplementary Table 11. Methylation analysis in under-annotated regions 33

Supplementary Notes **34**

Supplementary Note 1: Genometric correlation analysis of DIP-seq datasets 34

Supplementary Note 2: In vitro activity and substrate specificity of N4CMT 35

Supplementary Note 3: Gene transcription and DNA modifications 37

Supplementary Note 4: Methylomes and small RNAs 39

Supplementary Discussion **40**

Supplementary References **42**

SUPPLEMENTARY FIGURES

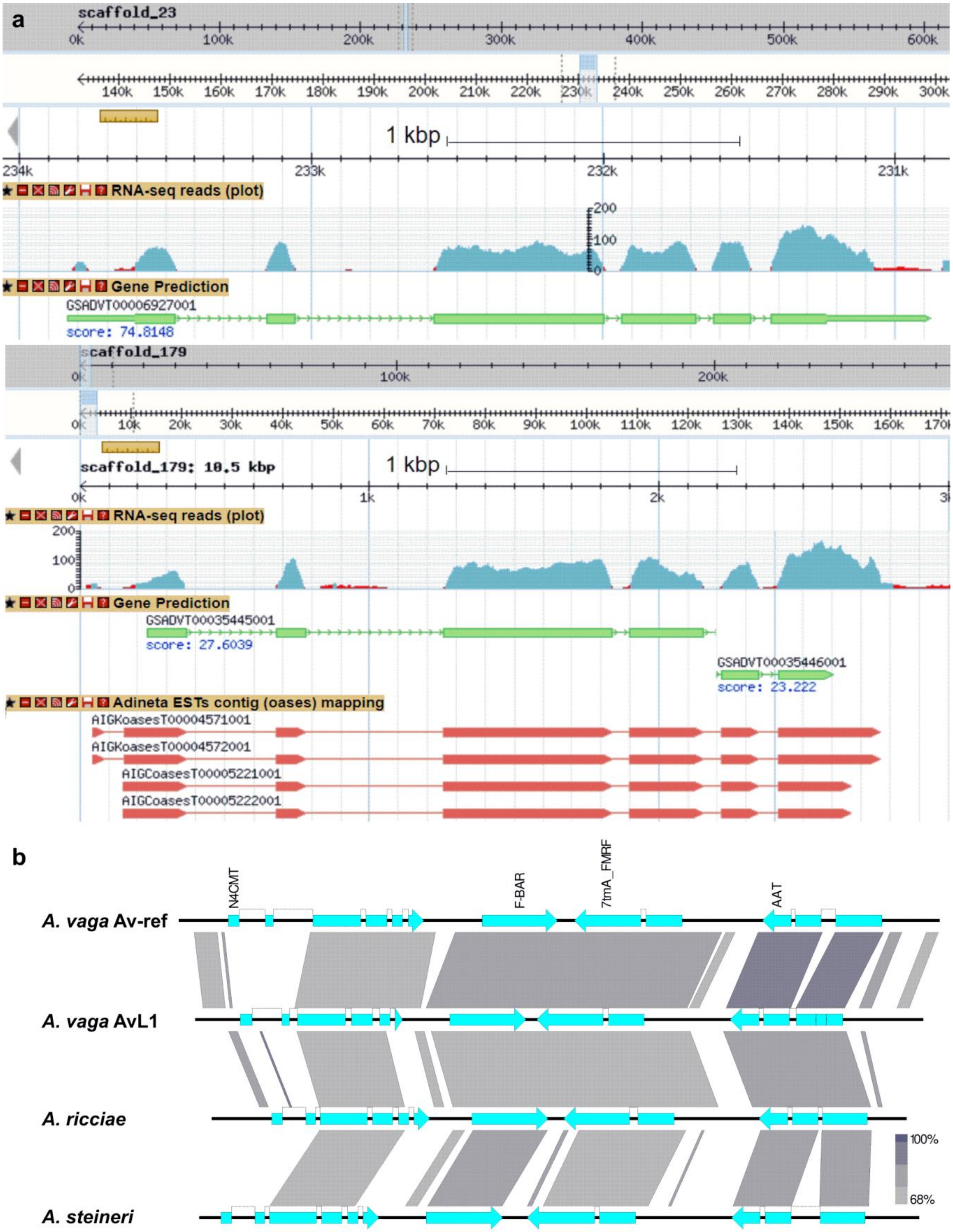

Supplementary Fig. 1. Genomic organization of the *N4CMT* gene in *A. vaga*.

a, Screenshots from the *A. vaga* genome browser (https://www.genoscope.cns.fr/adineta/cgi-bin/gbrowse/adineta) showing the RNA-seq reads, exon-intron structure, and EST contig mapping for two allelic scaffolds 23 and 179. The red color in RNA-seq plots denotes read coverage lower than 15. **b,** Synteny in N4CMT genomic environments in four species from the genus *Adineta*. Predicted metazoan genes on genomic scaffolds are shown by blue arrows. Regions of homology are gray-shaded, and the intensity of shading corresponds to nucleotide sequence identity (%) as indicated. BLASTN output was used for pairwise comparison of scaffolds, plotting similarities with e-values < 0.001. The figure was generated with Easyfig 2.2.2 [1]. Genbank accession numbers: HG380780.1 [https://www.ncbi.nlm.nih.gov/nuccore/HG380780.1] (*A. vaga*), OEOB01004975.1 [https://www.ncbi.nlm.nih.gov/nuccore/OEOB01004975] (*A. ricciae*), CAJNOE010000885.1 [https://www.ncbi.nlm.nih.gov/nuccore/CAJNOE010000885] (*A. steineri*).

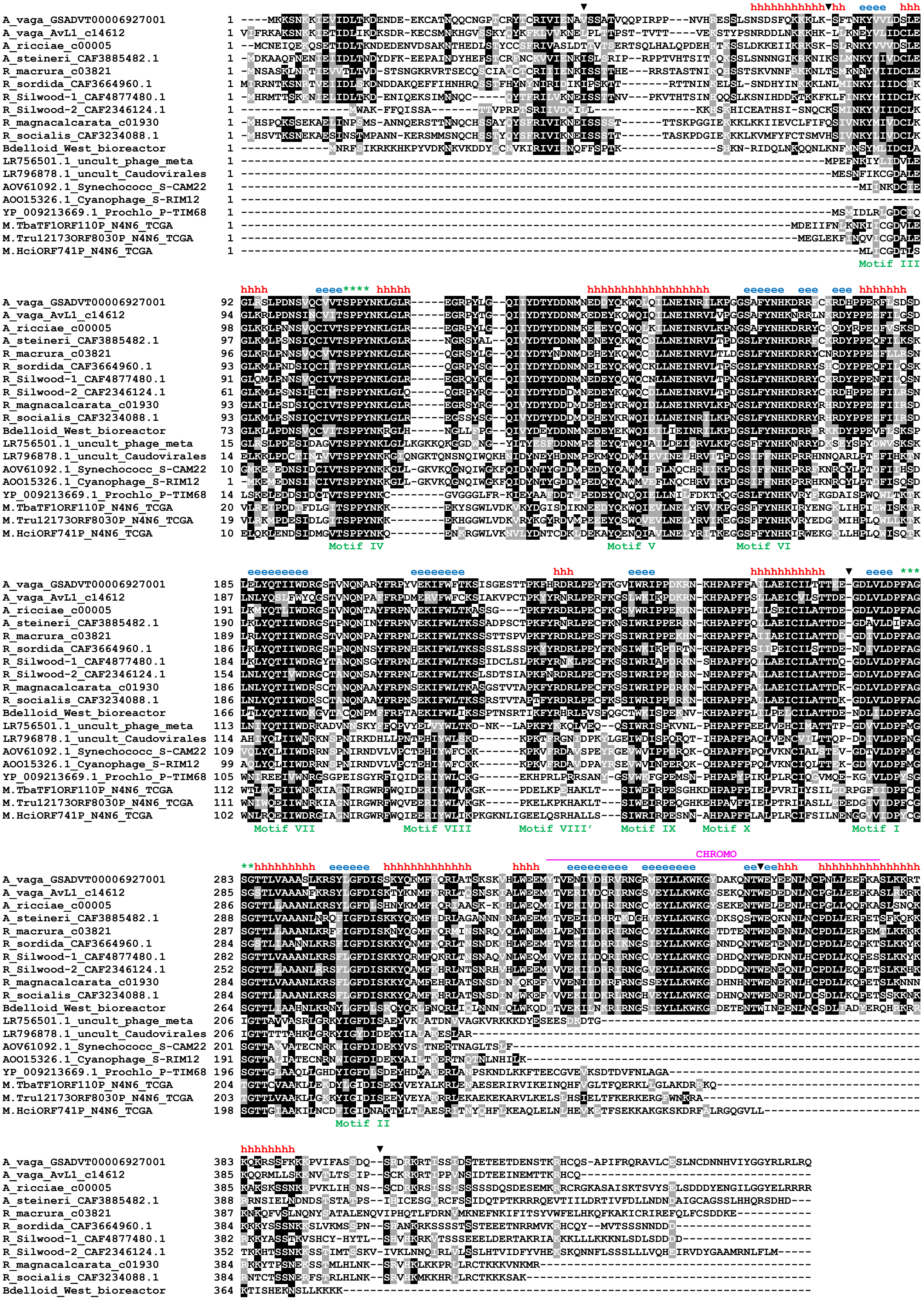
A_vaga_GSADVT00006927001
A_vaga_AvL1_c14612
A_ricciae_c00005
A_steineri_CAF3885482.1
R_macrura_c03821
R_sordida_CAF3664960.1
R_Silwood-1_CAF4877480.1
R_Silwood-2_CAF2346124.1
R_magnacalcarata_c01930
R_socialis_CAF3234088.1
Bdelloid_West_bioreactor
LR756501.1_uncult_phage_meta
LR796878.1_uncult_Caudovirales
AOV61092.1_Synechococc_S-CAM22
AOO15326.1_Cyanophage_S-RIM12
YP_009213669.1_Prochlo_P-TIM68
M.TbaTF1ORF110P_N4N6_TCGA
M.Tru12173ORF8030P_N4N6_TCGA
M.HciORF741P_N4N6_TCGA
Motif III
Motif IV
Motif V
Motif VI
Motif VII
Motif VIII
Motif VIII'
Motif IX
Motif X
Motif I
Motif II
CHROMO

Supplementary Fig. 2. **Amino acid sequence alignment of N4CMT from ten bdelloid species**. Shown are the full-length predicted N4-MTases from 9 bdelloid species (Supplementary Table 2) and a member of the family Habrotrochidae assembled from metagenomic data[2]; phages with GenBank accession numbers; and bacteria with REBASE ID's. Shading of conserved residues was done with Boxshade 3.21. Conserved MTase motifs (green) are shown on the bottom. Conserved intron positions in bdelloids (▼), secondary structure elements in AvCMT predicted by AlphaFold2[3], MTase catalytic residues (*) and the chromodomain moiety (purple) are marked on the top.

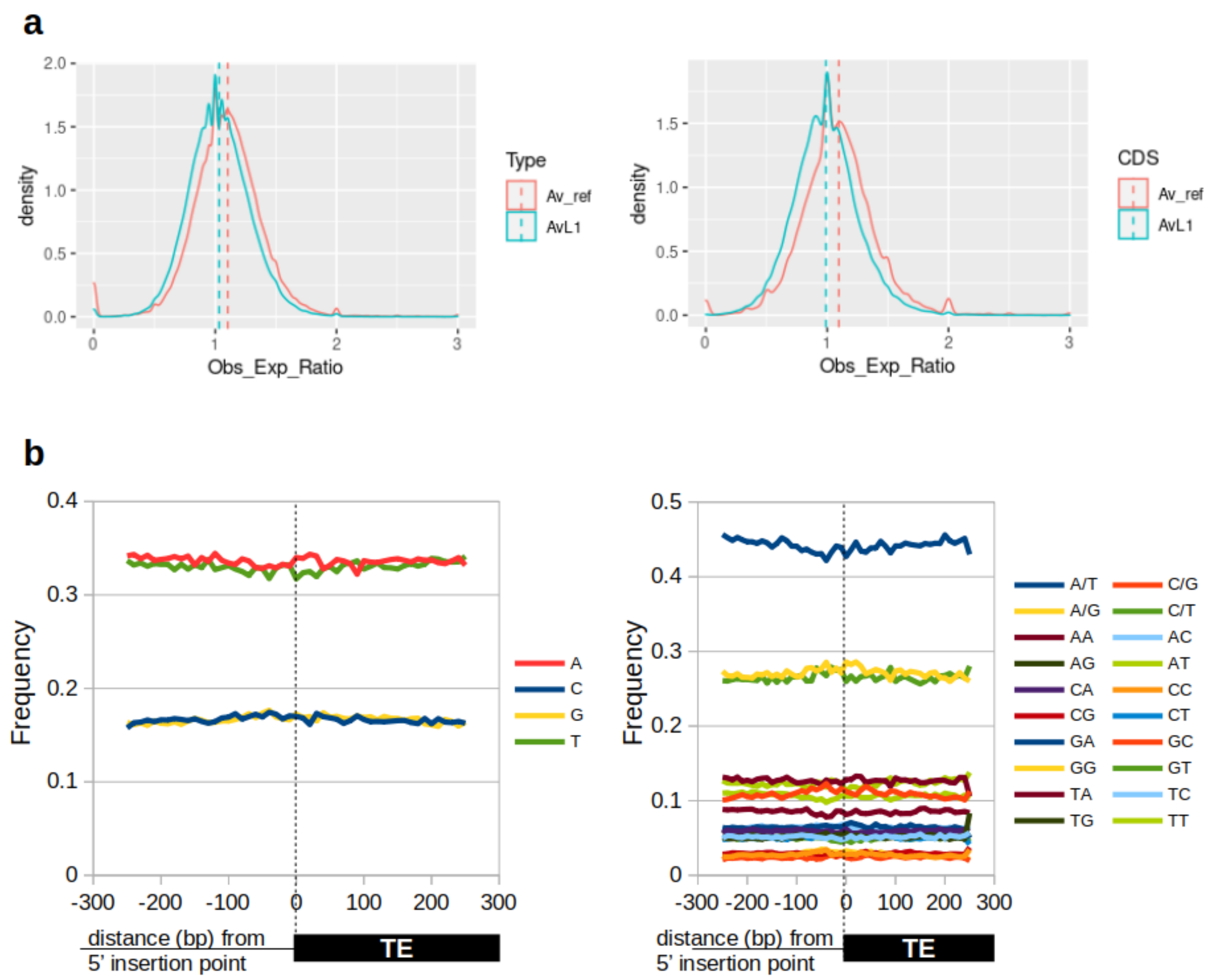

Supplementary Fig. 3. **Base and dinucleotide composition features in *A. vaga***.

a, Distribution of the observed/expected ratio of CpG dinucleotide frequency in Av-ref and AvL1 assemblies in a 1-kb sliding window (left panel) and in CDS regions (right panel). Its mean value, 1.103 and 1.032 for Av-ref and AvL1, respectively, indicates the lack of pronounced 5mC deamination signatures in gDNA. CDS ratio was calculated per gene, with 1.096 and 0.990 as mean CpG obs/exp values for Av-ref and AvL1, respectively.

b, Nucleotide and dinucleotide composition frequencies across AvL1 TE annotations and 5' upstream regions.

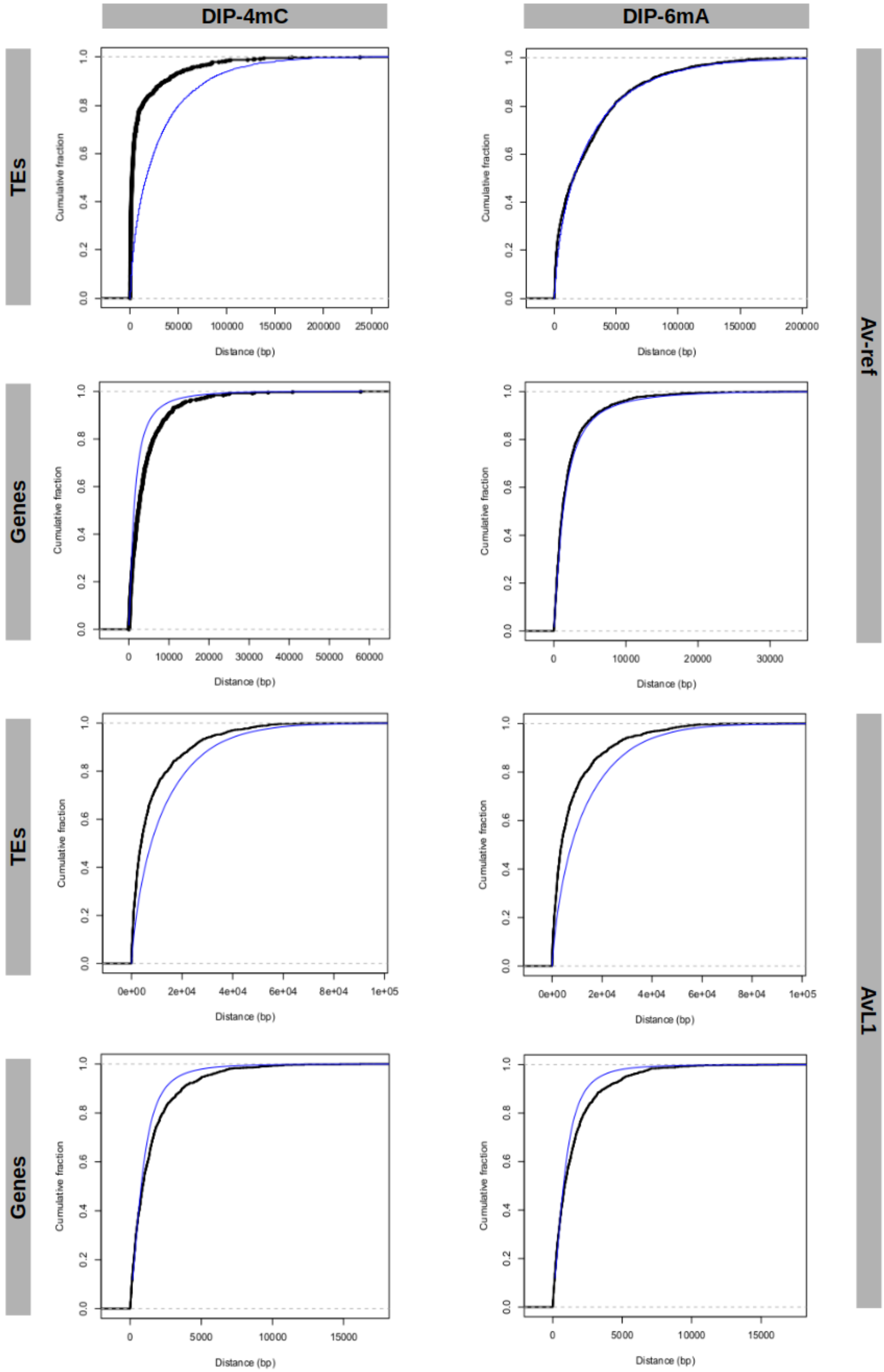
DIP-4mC
DIP-6mA
TEs
Genes
TEs
Genes
Av-ref
AvL1
Cumulative fraction
Distance (bp)

Supplementary Fig. 4. Genometric correlations in *A. vaga* (Av-ref and AvL1 strains).

Graphical output for the absolute distances between TE and gene annotations with DIP-seq methylation peaks detected across the genomes. For each assembly, summary results for all contigs together are displayed in four panels. Observed distributions are shown by black lines, while expected (distribution if there is no association) are blue. The output shows an absolute/relative positive (i.e., small distance between) correlation (black line over blue line) for DIP-seq 4mC and transposon annotations in both genomes (Av-ref and AvL1), while, for instance, 4mC and genes showed a negative (black line under blue line) correlation (i.e., spatial separation).

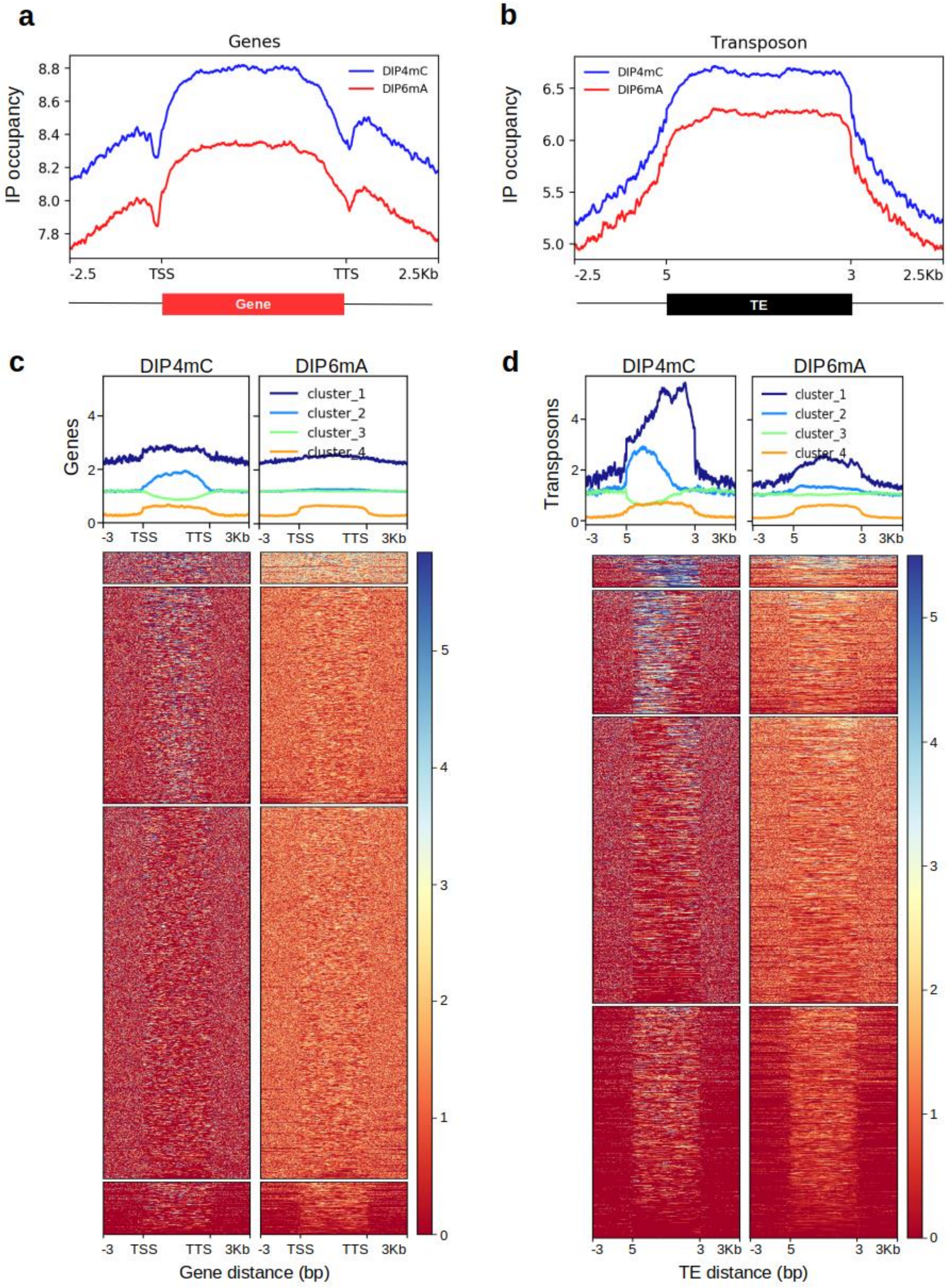
a
Genes
IP occupancy
DIP4mC
DIP6mA
-2.5
TSS
TTS
2.5Kb
Gene
b
Transposon
TE
c
DIP4mC
DIP6mA
Genes
cluster_1
cluster_2
cluster_3
cluster_4
Gene distance (bp)
d
Transposons
TE distance (bp)

Supplementary Fig. 5. DIP-seq profiles near genes and transposons in AvL1.

a-b, Distribution of 4mC and 6mA DIP-seq reads around gene (**a**) and transposon (TE) (**b**) annotations in AvL1 using meta-profile plots, with coverage shown within and in the proximity of annotated features. IP occupancy is represented in 25-bp bins within 2.5 kb upstream and downstream. The body size feature, representing genes or TEs, is automated and normalized as meta-profile.
c-d, Profiles (top) and heat maps (bottom) in AvL1 showing DIP-seq reads for 4mC and 6mA over features divided into four clusters (deepTools2--kmer 4). (**c**), Gene regions with TSS (transcription start site) and TTS (transcription termination site) and their vicinity (±2.5 kb). (**d**), TE annotations within 5' (5) and 3' (3) sites and near insertion points (± 2.5 kb). Heatmap color bars represent H3K enrichment (RPGC normalized read density).

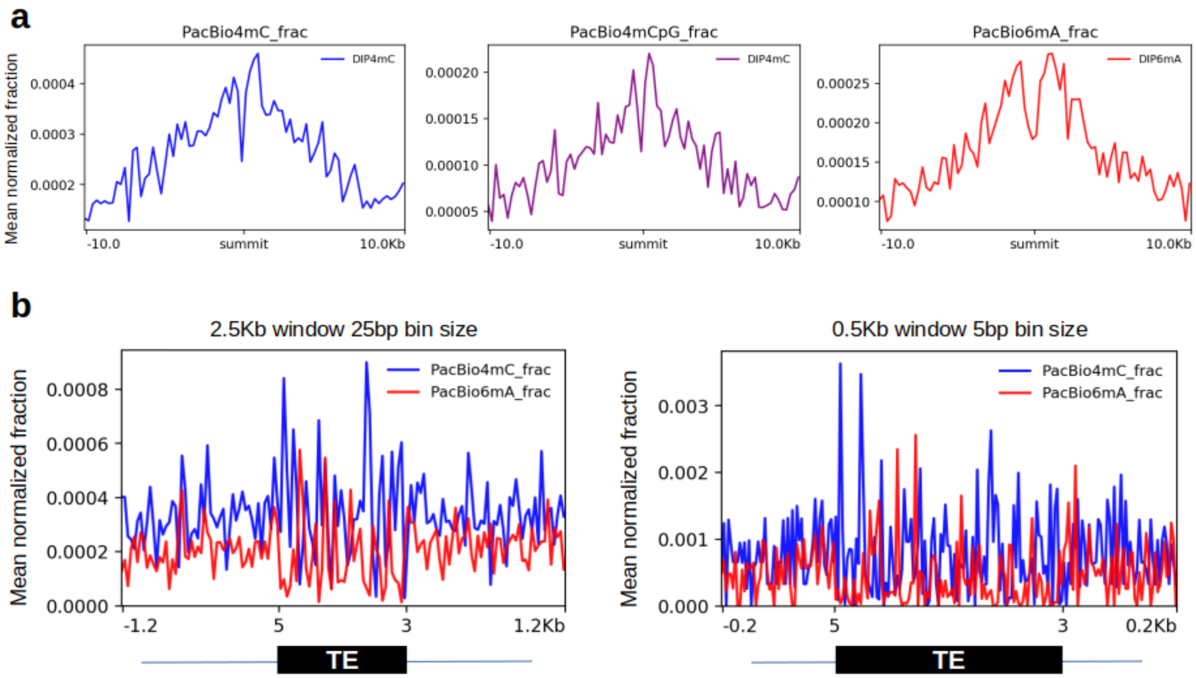

Supplementary Fig. 6. Fractional methylation profiles in *A. vaga*.
a, Distribution profiles of 4mC, 4mCpG and 6mA SMRT-seq methylation marks normalized by fraction values around DIP-seq 4mC (left and center) and 6mA (right) peak summits in AvL1.
b, Distribution profile of 4mC and 6mA SMRT-seq methylation marks normalized by fraction values across TE annotations (metaprofiles) for two different window and bin sizes in AvL1.

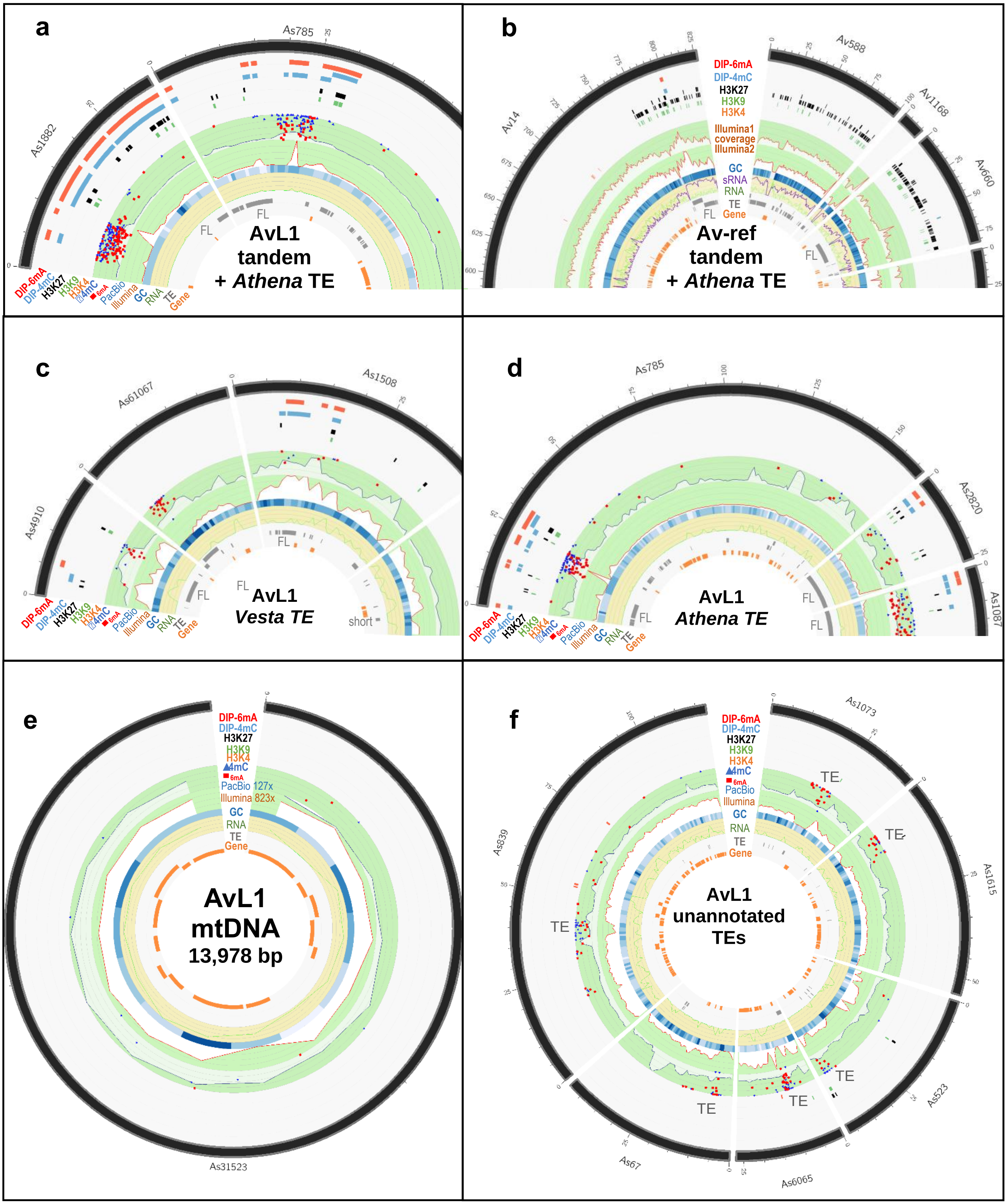
a
AvL1
tandem
+ Athena TE
b
Av-ref
tandem
+ Athena TE
c
AvL1
Vesta TE
d
AvL1
Athena TE
e
AvL1
mtDNA
13,978 bp
f
AvL1
unannotated
TEs

Supplementary Fig. 7. **DNA and histone modifications on selected contigs.**

a,b, contigs containing tandem repeats; **c,d**, contigs containing retrotransposable elements; **e**, mtDNA contig; **f**, contigs with unannotated TE copies. Circos plots of Av-ref (Av) and AvL1 (As) profiles show seven layers with annotations, sequencing coverage, methylation sites, and DIP/ChIP peaks. Inside to outside: grey bars, TEs; orange bars, genes; green line, RNA-seq coverage for Av-ref and AvL1 transcriptomes; purple line, small RNA coverage in Av-ref. Blue histogram, % G+C (light-blue, low GC; dark-blue, high GC). In DNA sequencing coverage plots, red and blue represent coverage by Illumina and PacBio reads, respectively; in (b), Illumina 1 and Illumina 2 tracks denote two Av-ref Illumina libraries, with 862 bp and 450 bp insert sizes, respectively. AvL1 PacBio layer shows DNA methylation sites for 4mC (blue triangles) and 6mA (red squares), with height in the ring showing methylation fraction (on a scale from 0 to 1). Histone methylation ChIP-seq peaks for H3K4me3, H3K9me3 and H3K27me3 are shown by orange, green and black bars, respectively. Blue and red bars, DIP-seq 4mC and 6mA peaks. Contig/scaffold ideograms are plotted as black bars, with labels for AvL1 (As) and Av-ref (Av) showing contig/scaffold numbers from source assemblies AvL1 (this study) and GCA_000513175, respectively. Label ticks are distanced at 5 kb. Coverage layers were calculated with 1-kb sliding window.

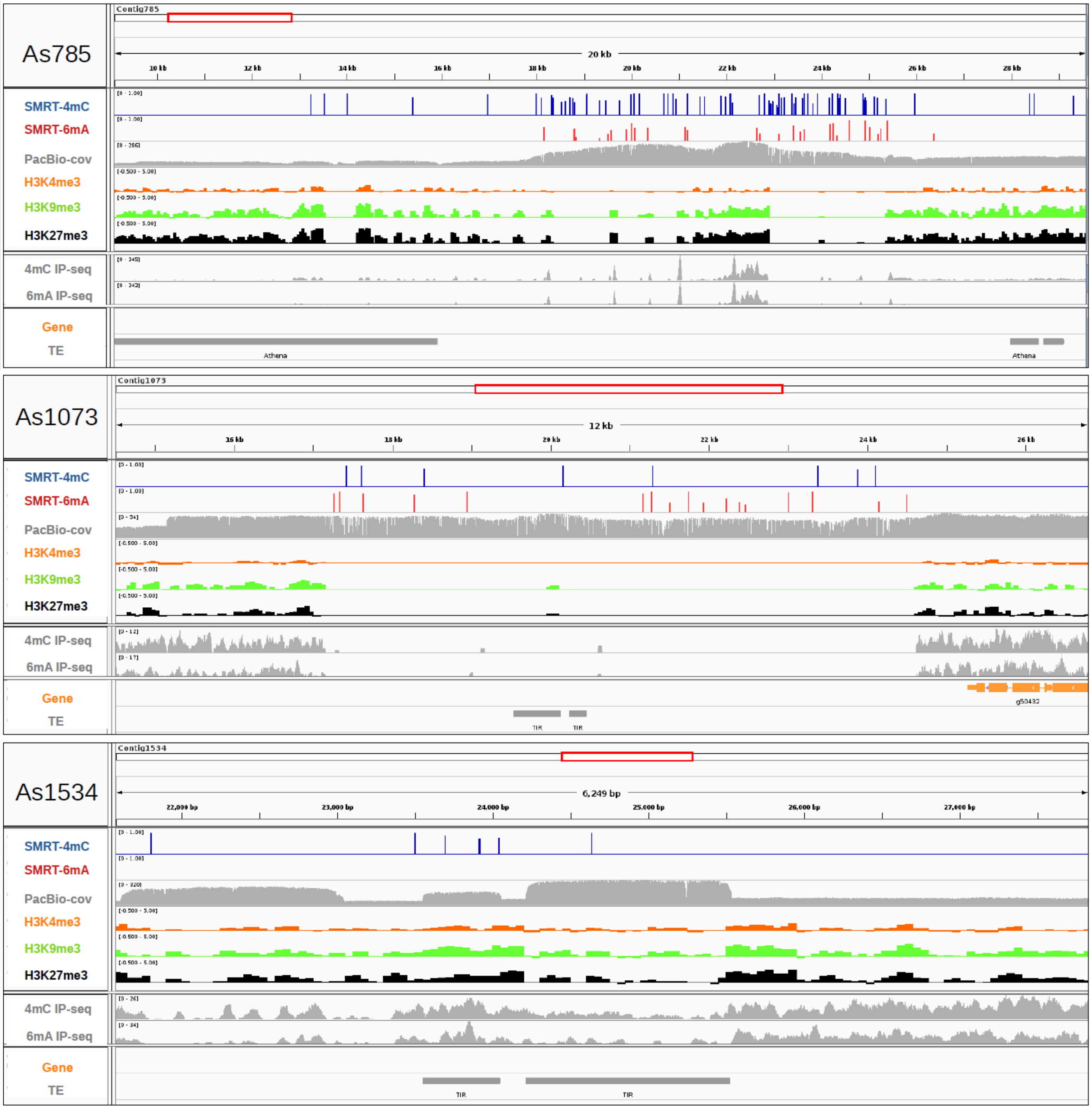

Supplementary Fig. 8. IGV browser views of DNA and histone methylation on selected AvL1 contigs.

IGV browser tracks are shown as follows: SMRT-seq methylation fraction (from 0 to 1) for 4mC (blue) and 6mA (red); PacBio read coverage (gray); ChIP-seq ratios (log2) for H3K4me3 (orange), H3K9me3 (green) and H3K27me3 (black); IP-seq coverage signal for 4mC and 6mA (gray). Gene and transposon (TE) annotations are represented in orange and grey, respectively. In contig As785, the highest methylation density is observed over tandem repeats shown in Supplementary Fig. 7a, which are located between *Athena* retroelements. Contig As1073 contains a polymorphic insertion of active *Chapaev* DNA TE present only in PacBio, but not Illumina, reads, which show methylation over TE in SMRT-seq tracks. Contig As1534 shows an unannotated DNA TE displaying 4mC but not 6mA marks (Supplementary Table 11).

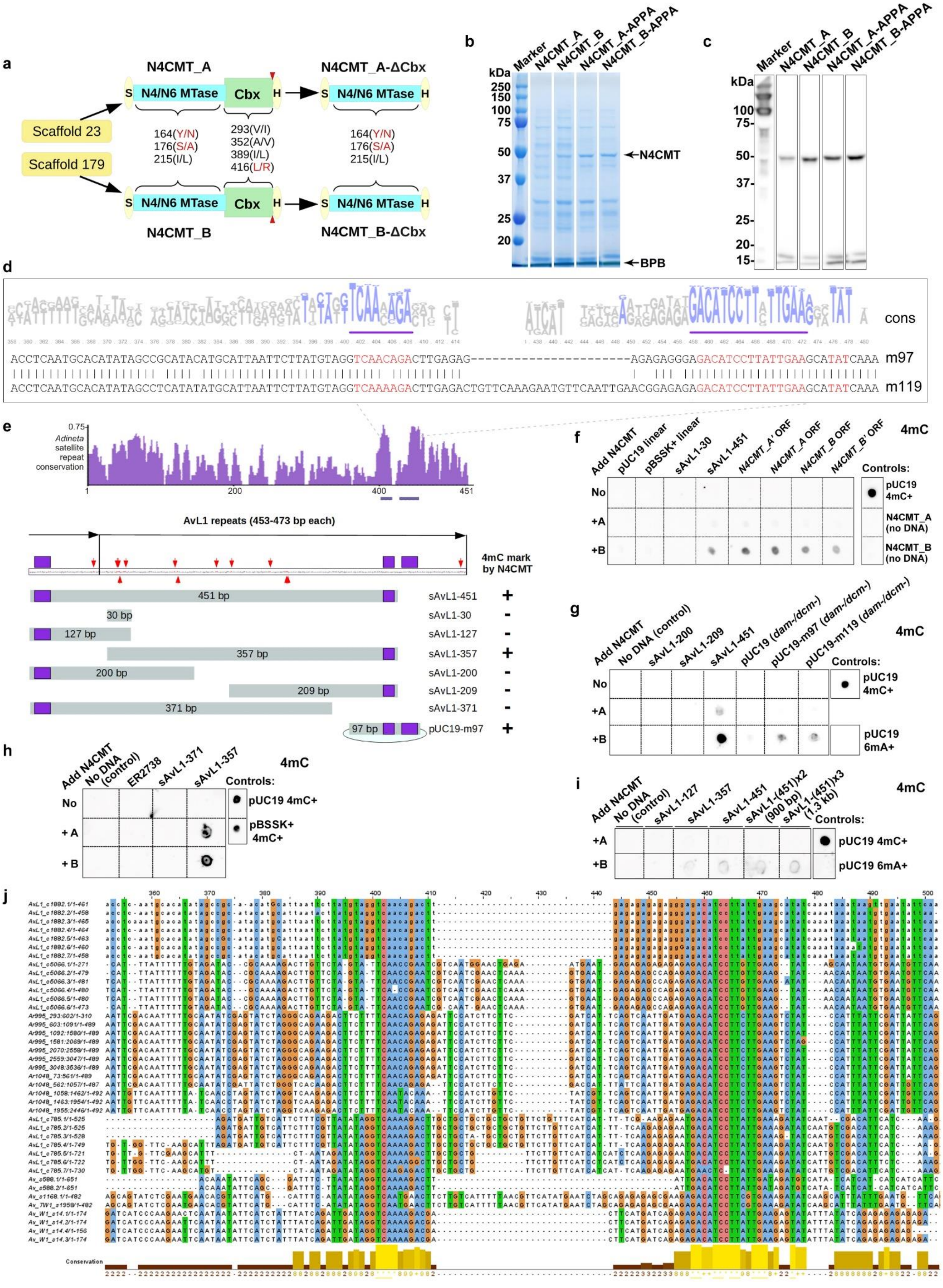
a
N4CMT_A
N4CMT_A-ΔCbx
N4/N6 MTase
Cbx
Scaffold 23
Scaffold 179
164(Y/N)
176(S/A)
215(I/L)
293(V/I)
352(A/V)
389(I/L)
416(L/R)
N4CMT_B
N4CMT_B-ΔCbx
b
Marker
N4CMT_A
N4CMT_B
N4CMT_A-APPA
N4CMT_B-APPA
kDa
250
150
100
75
50
37
25
20
N4CMT
BPB
c
kDa
100
75-
50-
37-
25-
20-
15-
d
cons
m97
m119
e
0.75
Adineta satellite repeat conservation
1
200
400
451
AvL1 repeats (453-473 bp each)
4mC mark by N4CMT
451 bp
sAvL1-451
30 bp
sAvL1-30
127 bp
sAvL1-127
357 bp
sAvL1-357
200 bp
sAvL1-200
209 bp
sAvL1-209
371 bp
sAvL1-371
97 bp
pUC19-m97
f
4mC
Add N4CMT
pUC19 linear
pBSSK+ linear
sAvL1-30
sAvL1-451
N4CMT_A' ORF
N4CMT_A ORF
N4CMT_B ORF
N4CMT_B' ORF
Controls:
No
+A
+B
pUC19 4mC+
N4CMT_A (no DNA)
N4CMT_B (no DNA)
g
4mC
Add N4CMT
No DNA (control)
sAvL1-200
sAvL1-209
sAvL1-451
pUC19 (dam-/dcm-)
pUC19-m97 (dam-/dcm-)
pUC19-m119 (dam-/dcm-)
Controls:
pUC19 4mC+
pUC19 6mA+
h
4mC
Add N4CMT
No DNA (control)
ER2738
sAvL1-371
sAvL1-357
Controls:
pUC19 4mC+
pBSSK+ 4mC+
No
+ A
+ B
i
4mC
Add N4CMT
No DNA (control)
sAvL1-127
sAvL1-357
sAvL1-451
sAvL1-(451)x2 (900 bp)
sAvL1-(451)x3 (1.3 kb)
Controls:
+A
+B
pUC19 4mC+
pUC19 6mA+
j
Conservation

Supplementary Fig. 9. **N4CMT expression, purification, and activity on different substrates**.

a, Diagram of recombinant N4CMTs used in this study. Amino acids differing between two variants amplified from scaffold_23 and scaffold_179 are shown. S, S tag; H, His tag. Vertical red arrows indicate C-terminal Leu or Arg substitutions giving rise to additional recombinant protein variants. Non-conservative amino acid substitutions are in red.

b, SDS-PAGE of purified N4CMTs. Arrows indicate positions of recombinant N4CMT proteins and the electrophoresis migration front dye bromophenol blue (BPB).

c, Western blotting of the gel identical to (**b**) with anti-His tag antibody, showing the presence of recombinant proteins with the expected molecular mass. SDS-PAGE and Western blotting were performed every time when fresh N4CMT proteins were purified, and after freezing to monitor protein quality. Representative gels demonstrating protein integrity are presented.

d, Nucleotide sequence conservation in tandem repeats with high density of 4mC modifications in *A. vaga*. Interspecific conservation was determined from two *A. vaga* isolates plus the sibling species *A. ricciae*. Two bottom sequences show the inserts m97 and m119 which confer N4CMT substrate properties to the pUC vector; conserved motifs are highlighted in red.

e, Diagram of the *A. vaga* 4mC-rich 460-bp tandem repeat region and its derivatives. Red arrows, modified cytosines. Conserved motifs are in purple. The dsDNA fragments used in activity assays are depicted on the bottom, with pluses and minuses summarizing N4CMT activity on the corresponding substrates, as determined by immuno-dot blots with anti-4mC antibody.

f-i, Immuno-dot blots with anti-4mC antibody for different substrates treated with recombinant N4CMT allozymes in vitro. Positive control, 100 ng *M.Bam*HI-treated pUC19. In (**f**), 500 ng of each DNA was loaded per dot, except for sAvL1-30 (6 μg). In (**g**), 1 μg of pUC19 plasmids (grown in *dam-/dcm- E. coli* strain) was loaded per dot. Linear dsDNA fragments (sAvL1-200, sAvL1-209, sAvL1-451) were equalized to 600 ng. In (**h**), 500 ng of each linear dsDNA (sAvL1-371, sAvL1-357) was loaded per dot and 400 ng of ER2738 gDNA was used. 100 ng of *M.Bam*HI-treated pBluescript SK+ (pBSSK+4mC+) was used as an additional positive control. In (**i**), sAvL1-451 repeats (1, 2 and 3 repeat units as labeled) were loaded at 600 ng per dot. sAvL1-127 and sAvL1-357 DNA amount was equalized to 2 pmol, which is equal to 600 ng used for sAvL1-451, to neutralize the amount of cytosines available for methylation.

j, Alignment of conserved regions in tandem repeat units from *A. vaga* (Av, AvL1) and *A. ricciae* (Ar) used to build the consensus in (**e**), visualized in Jalview 2.11.1.3. Not more than seven repeat units from each contig are shown. The yellow color on the bottom shows the high degree of conservation.

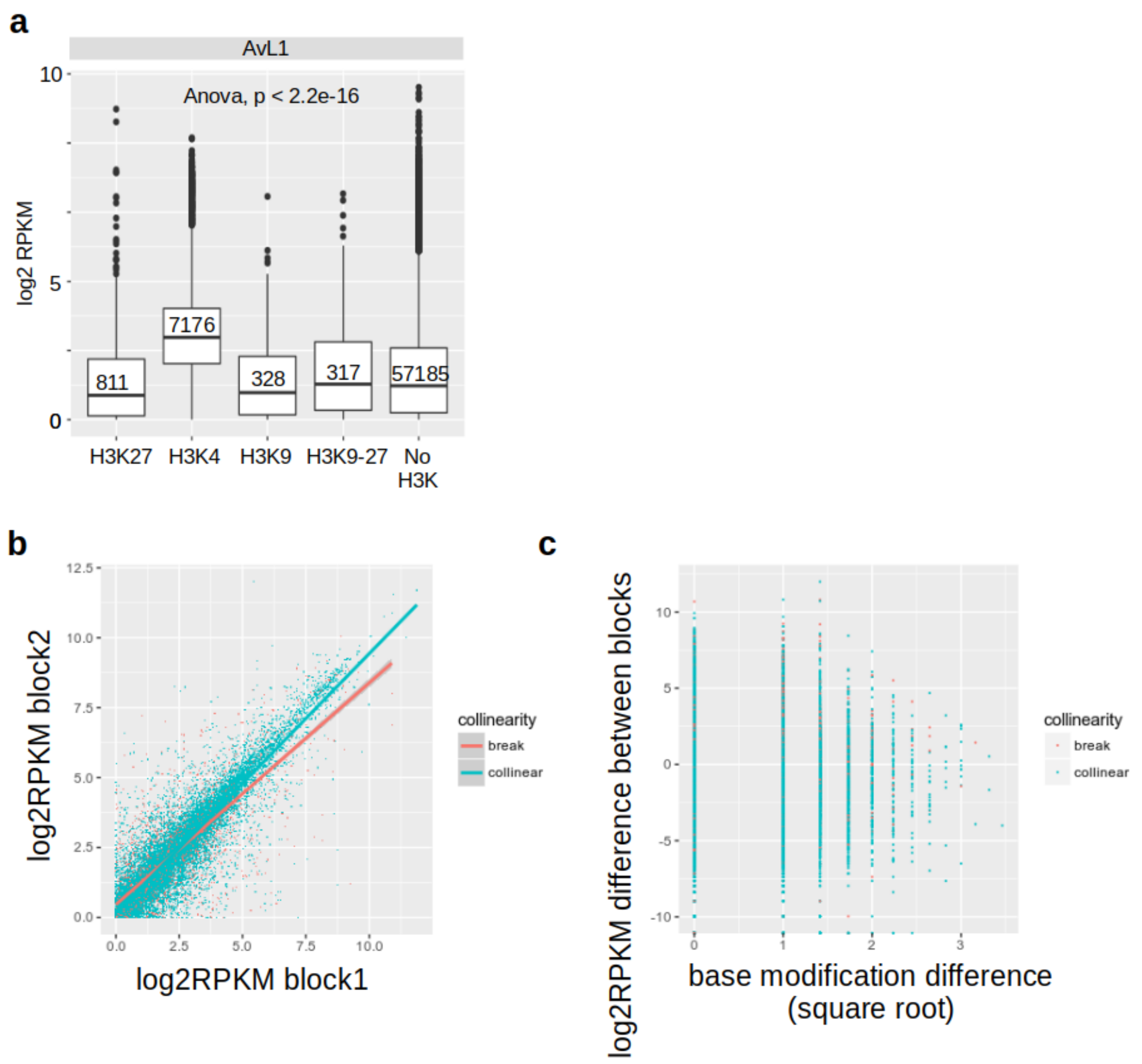

Supplementary Fig. 10. Comparisons of AvL1 gene transcription in ChIP-seq peaks and in collinear blocks.

a, Box plot showing AvL1 gene expression levels (log_2RPKM) associated with co-localized H3K4me3, H3K9me3, H3K27me3, and H3K9-27me3 marks or without histone marks. ANOVA analysis shows significant differences in expression (one-sided ANOVA, Df = 4, F = 1331), with genes associated with the H3K4me3 mark displaying the highest RPKM (reads per kilobase per million mapped reads). Box plots indicate the first and third quartiles; line, the median; single points as outliers. Error bars are calculated as the standard deviation of two technical replicates.

b, Transcription in collinear blocks. Points represent collinear blocks of genes, plotted based on the transcription values (log_2RPKM) on block1 (X-axis) and transcription on block2. Syntenic blocks are differentiated between two groups: collinear (homologous genes with low Ks values) and blocks in which collinearity has been broken (homeologous genes with high Ks values).

c, Base modification versus transcription differences. X-axis represents the difference between blocks in the number of detected SMRT base modifications (square root), and Y-axis represents the absolute difference in log_2RPKM between them.

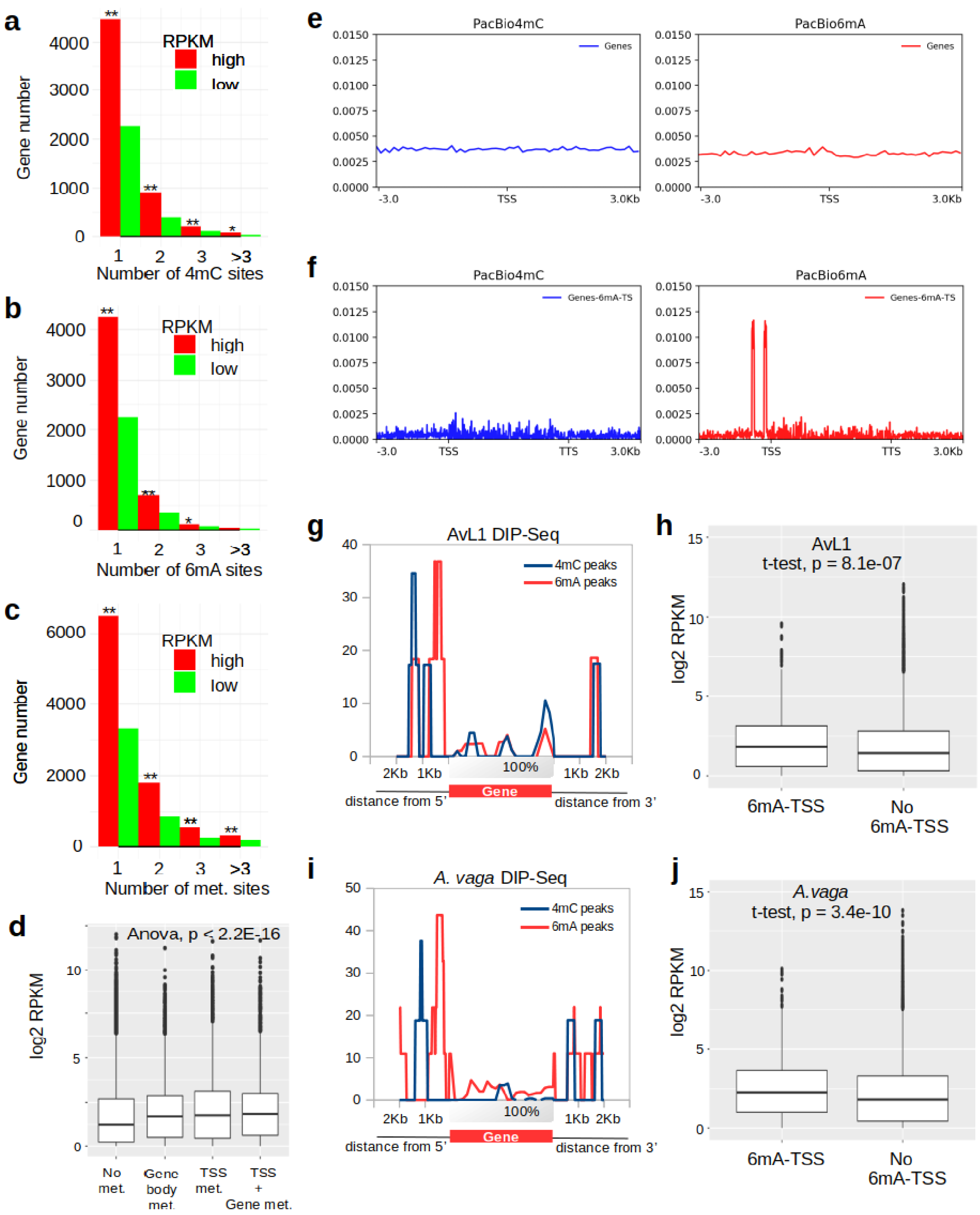
a
RPKM
high
low
Gene number
Number of 4mC sites
b
RPKM
high
low
Gene number
Number of 6mA sites
c
RPKM
high
low
Gene number
Number of met. sites
d
Anova, p < 2.2E-16
log2 RPKM
No met.
Gene body met.
TSS met.
TSS + Gene met.
e
PacBio4mC
Genes
PacBio6mA
Genes
-3.0
TSS
3.0Kb
f
PacBio4mC
Genes-6mA-TS
PacBio6mA
Genes-6mA-TS
-3.0
TSS
TTS
3.0Kb
g
AvL1 DIP-Seq
4mC peaks
6mA peaks
2Kb 1Kb
100%
1Kb 2Kb
Gene
distance from 5'
distance from 3'
h
AvL1
t-test, p = 8.1e-07
log2 RPKM
6mA-TSS
No 6mA-TSS
i
A. vaga DIP-Seq
4mC peaks
6mA peaks
2Kb 1Kb
100%
1Kb 2Kb
Gene
distance from 5'
distance from 3'
j
A.vaga
t-test, p = 3.4e-10
log2 RPKM
6mA-TSS
No 6mA-TSS

Supplementary Fig. 11. **Correlation between gene transcription and SMRT modification marks**.
a-c, Numbers of methylated genes with 1, 2, 3 or more (>3) marks for 4mC (**a**), 6mA (**b**) and both combined (**c**) are plotted with the RNA-seq transcription profiles (high for RPKM≥1, low for RPKM<1). Asterisks show statistically significant differences in the numbers of methylated genes between high and low RPKM levels (χ^2 test, **p<10-4, *p<0.05). Exact p-values: (a) 1, 4.538E-56; 2, 2.171E-18; 3, 1.332E-03; >3, 4.059E-02; (b) 1, 4.111E-40; 2, 4.720E-08; 3, 1.339E-02; >3, 7.049E-01; (c) 1, 8.980E-80; 2, 6.809E-31; 3, 1.152E-10; >3, 3.022E-05.
d, Boxplot comparing expression ($\log_2$RPKM) of genes with no methylation; methylation in the gene body; around TSS (2 kb upstream); and both methylated regions combined (TSS plus body). ANOVA analysis shows differences in expression of 4mC (ANOVA one-sided with Tukey's *post hoc*; p-val < 2.2E-16).
e, f, 4mC and 6mA SMRT-seq methylation in the final gene set (n=65,934) (**e**) and clusterized for 6mA signal in the TSS region (n=1212) (**f**).
g-h, Distribution of 4mC and 6mA DIP-seq peaks (**g**) and RPKM (**h**) around AvL1 genes with 6mA modification (n=1212) showing coverage of peak deposition within and near genes. In (**h,j**), genes marked with 6mA around TSS exhibit higher expression than genes without it.
i-j, Distribution of 4mC and 6mA DIP-seq peaks (**i**) and RPKM (**j**) in Av-ref genes homologous to AvL1 genes in (**e**-**h**). In (**g**, **i**), peak coverage is shown in 25-bp bins within ±2 kb. The body size feature, representing genes, is automated and normalized as a meta-profile (0-100% of body length). In (**h**, **j**), boxplot compares the median and interquartile range of RPKM expression levels. The p-values were calculated by a two-tailed Student's t-test.
In (**d**, **h**, **j**), box represents the first and third quartiles; line, the median; single points as outliers. Error bars are calculated as the standard deviation of three biological replicates (Av-ref) or two technical replicates (Av-L1). RPKM, reads per kb per million mapped reads.

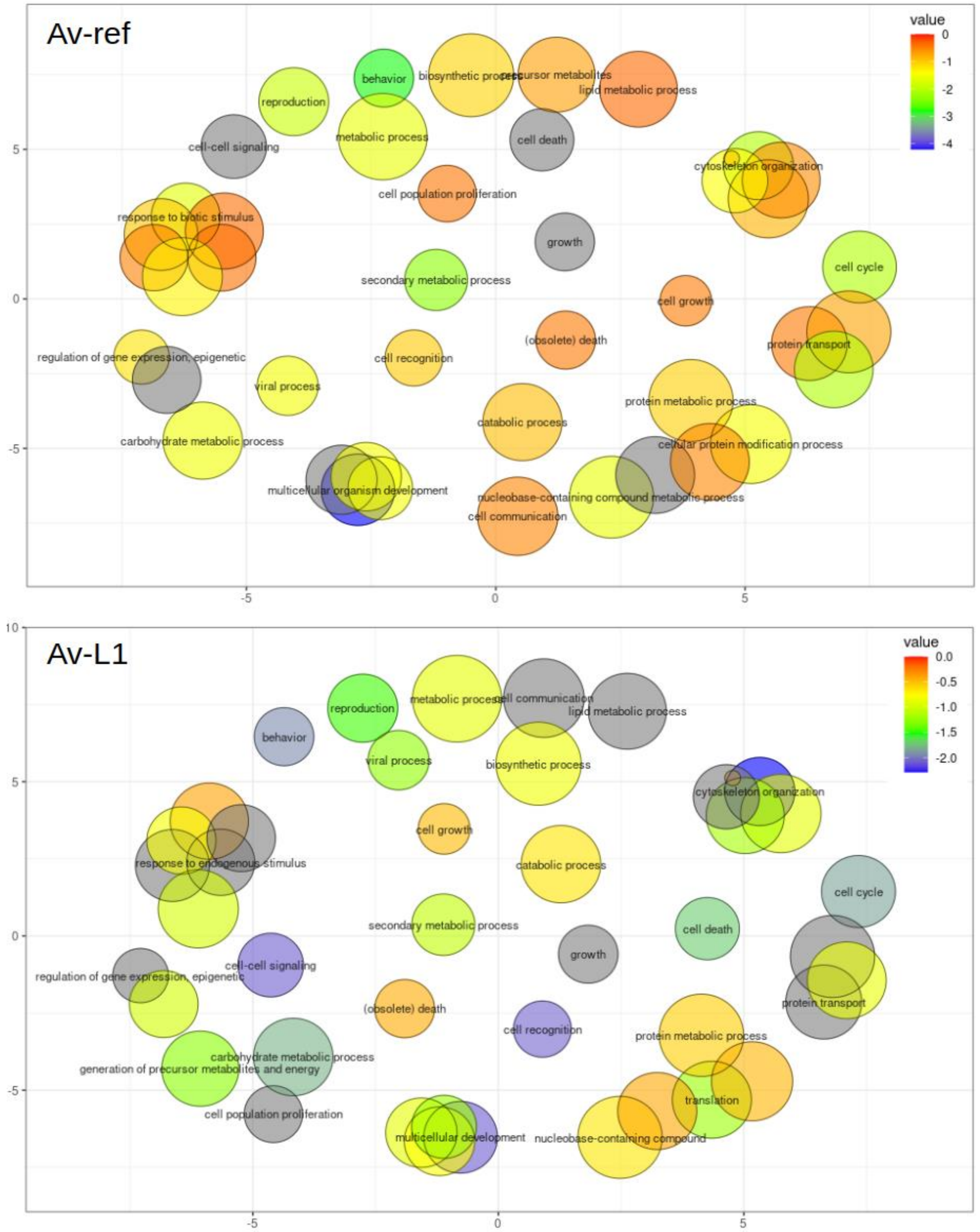

Supplementary Fig. 12. Gene ontology (GO) enrichment of biological processes in 6mA-methylated orthologs from Av-ref and AvL1 strains. In the scatterplots, each bubble represents a significantly enriched term in a two-dimensional space derived by applying multidimensional scaling to a matrix of the GO terms' semantic similarities (Methods). Bubble color is calculated as |Log10_p-value| of two-sided Fisher's exact test, as indicated in the color bar. Bubble size indicates the frequency of the GO term in the underlying GOA (GO annotation) database (bubbles of more general terms are larger). Source data are provided in Source Data 1.

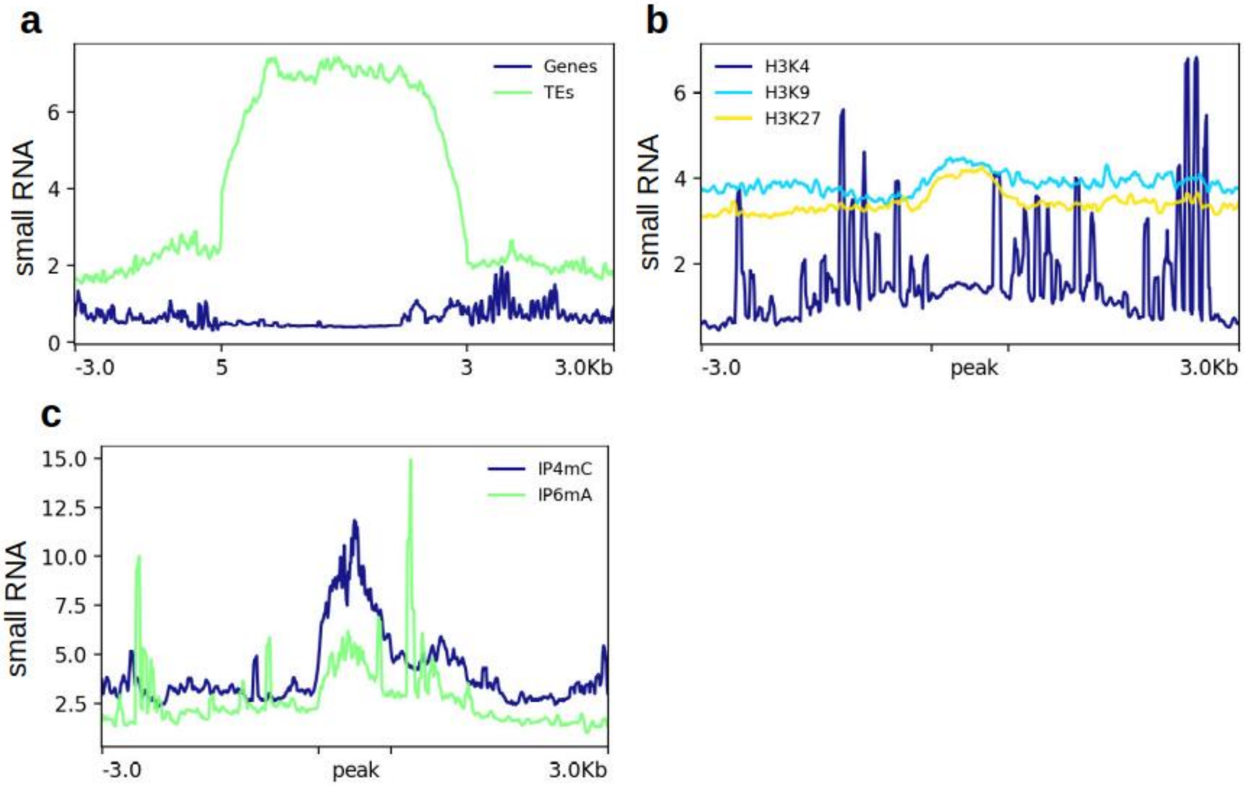

Supplementary Fig. 13. Association of small RNAs with histone and DNA methylation.

a-c, Distribution of sRNA with respect to genes and TEs (**a**), H3K4, H3K9, H3K27 ChIP-seq peaks (**b**), and IP4mC, IP6mA DIP-seq peaks (**c**). Relative fold enrichment is shown as reads per genomic context (RPGC normalization). In gene and TE profiles, regions in the map comprise gene bodies (5 for TSS and 3 for TTS) or TE bodies (5 for the 5'-boundary and 3 for the 3'-boundary) with ±3 kb flanks. Peak profiles are represented by peak body flanked by ±3 kb.

```
a cd01395 HMT_MBD           # # #                 # #         #  #   #
Avaga314   394 PFLLPf-DCRWSIVDSKPRg--------------------YRTPCC-RTLYSLDDIEQYLyrt----qskLSIKYFVD 445  Adineta vaga
Avaga19b   394 PFLLPf-KCQWSIVDGKPRg--------------------YRTPCH-RTLYSLDDIEKYLfrt----dskLSIKFFID 445  Adineta vaga
Avaga150   247 FLLIPl-NYGWKRQIRNDLg-----------------TIVYISPCG-QMFTDGERLHAFLyqt----eslLSIRQFTF 301  Adineta vaga
Avaga176   245 FLLIPl-NQGWKRQIRNDLg-----------------TIVYISPCG-QMFTDADRLQSHLfqm----nslLNIRQFSF 299  Adineta vaga
Avaga19a   351 PYTLPf-SYGWRYLYIDRYakgyyskkpsthktvSRVNYLYRSPCG-RSLLTLDEVENYLfet----nskLTIKFFID 422  Adineta vaga
Avaga135   550 PYLKPi-ACQWTILETIRIkkandv-----rvknIRAILIYRAPCG-RKLSNEAQVDNYLhet----qsrLSIELFVF 616  Adineta vaga
Avaga90    540 PFLKPf-ACHWTMLDNVRVrkagdv-----rlklARSTFIYCSPCG-RKLVSQGQLDNYLhet----kskLTIELFVL 606  Adineta vaga
RNA32532   827 PLLIPv-LHGWQRHIGNISknp---------ttmTKKFIRYVTPCG-RMLRSTAEIDHYLylt----nskLTIDMFTT 889  B. plicatilis
Q96T68     164 PLQLPi-KCHFQRRHAKTNsh------------sSALHVSYKTPCG-RSLRNVEEVFRYLlete---cnfLFTDNFSF 224  human SETDB2
AAN61106   183 PLQLPi-SCDFQRCHLKINsp------------dDLSHILYKAPCG-RSLRDYDEVHSYLtetg---chfLAVDNFSF 243  X. laevis CLLD8
CAF97873   621 PLLMPl-LYEFRRMTGRRKvn-----------rkMSFHVIYKAPCG-LCLRNMSEIQHYLfqtn---cdfIYLEMFCL 682  T. nigroviridis
Q15047     599 PLLVPl-LYDFRRMTARRRvn-----------rkMGFHVIYKTPCG-LCLRTMQEIERYLfetg---cdfLFLEMFCL 660  human SETDB1
AAN71064   446 PLAKPl-LSGWERLVMRQK---------------TKKSVVYKGPCG-KSLRSLAEVHRYLrat----envLNVDNFDF 502  D. melanogaster
P34544     829 PIHTPl-LCGWRRIMYTMStgkk--------rgaVKKNIIYFSPCG-AALHQISDVSEYIhvt----rslLTIDCFSF 892  C. elegans met-2
CAE71274   782 PLHTPl-LCGWKRLKYTMHsgkk--------rasVKKVIIYRAPCG-KPLEKTSEIADYLrst----rsqLTIDCFSF 845  C. briggsae
XP_317488  318 LLARPl-ITGWERHKTAARg-------------qKKSVVLYRAPCG-RRLRNMAELHQYLrvt----dspLNVEHFDF 376  A. gambiae str. PEST
                          eeeeee                   eeeeee      ee  hhhhhhhhhhh                           1QK9

b cd01396 MeCP2_MBD         # # #                 # #         #  #   #
1QK9_MeCP2  18 MYDDPtLPEGWTRKLKQRKsgr----------saGKYDVYLINPQG-KAFRSKVELIAYFekvg---dtsLDPNDFDFtv-----tGRGSGSGC 92  human
AAM63666    29 PGDDNwLPPDWRTEIRVRTsgt----------kaGTVDKFYYEPITgRKFRSKNEVLYYLehgtpkkksvKTAENGDShse--hseGRGSARRQ 110 Arath
CAE05215    27 KIEPA3LPHGWLKEYRPRKnqs---------gsrVKGDTFYIDPTNmYEFRSQKDVQRYLe--------sGDVTNCVMiqnkrkmeDLHTARNQ 105 rice
NP_200746   76 PGDNW-LPPGWRVEDKIRTsga----------taGSVDKYYYEPNTgRKFRSRTEVLYYLehgts-krgtKKAENTYFnpd--hfeGQGSNRVT 155 Arath
1IG4_MBD1    5 WLDCPaLGPGWKRREVFRKsga----------tcGRSDTYYQSPTG-DRIRSKVELTRYLgp-------aCDLTLFDFkq-----gILCYPAPK 75  human
AAK40309   135 KPNIAqPPHGWERQIRIRGeg-----------gtKFADVYYTSPTG-RKLRSLVEVDRFLqenpehvaqgVTLAQFSFqip--rplRQDYVKKK 214 maize
NP_974851   57 WPSIPpIPTGWSRSVHIRSes------------tKFADVYYFPPSG-ERLRSSAEVQSFLdnhpeyvregVNRSQFSFqip--kplDDNYVKKR 135 Arath
BAB11480   120 KPNISrPPAGWQRLLRIRGeg-----------gtRFADVYYVAPSG-KKLRSTVEVQKYLndnseyigegVKLSQFSFqip--kplQDDYVRKR 199 Arath
T04569     108 KPGLPrTPRGFKRSLILRKd-------------ySKMDAYYITPTG-KKLKSRNEIAAFIdanq--dykyALLGDFNFtvp--kvmEETVPSGI 183 Arath
AAK40305    84 KPNIP-KPPPETERLVVMRgd------------ySKMDTYYVMPNG-KRARCAGDVDKFLeanpe-ykdrISASDFSFapp--kvvEETVSHNP 160 maize
               ee        eeeeee                    eeeeee      ee  hhhhhhhh                                   1IG4

c cd01397 HAT_MBD           # # #                 # #         #  #   #
Q9UIF8     548 ELRIPl-EYGWQRETRIRNfg-----------grLQGEVAYYAPCG-KKLRQYPEVIKYLsrng---imdISRDNFSFSAKIRVGDFYE 620  HomoBAZ2B
XP_317442  303 ELRVPl-EKGWRRETVIRGltr---------nghIKGDVYYYPPQS-VNKMKGMNQIQLYldqf--kpkdLSRDNFSFSAKAIVGTFLQ 378  A.gambiae
AAK00302   953 ELRVPl-ELGWKRETVIRGltk---------qgqIRGEVTYYAPGS-TTPLKSNGQVFAIleqq---psnLSRENFSFSARAIVGSFLQ 1027 D.mel TOU
Q9DE13     692 ELRVPl-EYGWQRETRIRNfg-----------grLQGEVAYFAPCG-KKLRQYPEVVKGVqw------clLKEEEVVPCIRAMEGRRGR 761  chicken
5AGQ_BAZ2  524 EVRLPl-QHGWRREVRIKKgs-----------hrWQGETWYYGPCG-KRMKQFPEVIKYLsrnl---vhsVRREHFSFSPRMPVGDFFE 596  HomoBAZ2A
AAN61105   423 QVCFPl-QHGWRREVRIKKgs-----------hrWQGETWYYAPCG-KRMKQFPEVIKYLskna---gpfVRREHFSFSPRMPVGDFYE 495  X. laevis
CAG04063    16 QVQFPl-QHGWKREIRVKKle-----------nrMKAETWYYTPCG-RRMKQFPEIIKYLkkht---dslVSREHFSFSPRMPVGDFYE 88   Tetraodon
CAF99785   176 ESGVGi-PGVAERNKNQNGgg------------rPQGEVAYYAPCG-KKLRQYPDVMKYLsrng---isgITRDNFSFSAKIRVGDFYE 247  Tetraodon
NP_498673  328 MLRLPl-QLGWRRQTCVRSias----------agVKGDVSYFAPCG-KKLSTYSEVVRYLtkns---ihyITRDNFLFNTKLVIGEFIV 401  C.elegans
RNA26940   163 KLRLPl-KIGYRRETLVREltn----------sgVKGDIIYYSPCG-RKLRNFQEIERYLykfya4knvqLTKENYTFSSKYIVGNYLL 143  B.plicati
Avaga76     77 LVRIPl-SRGWKRVTIVRAitr----------tgVRGDVSYYAPCG-RKLRSFQEIDRYLskkn---itdLDRSHFTFSSKVHIGHFHE 150  A. vaga
Avaga413    38 LVRIPl-NRGWKRITIVRAitr----------tgVRGDVSYYAPCG-RKLRSFQEIDRYLskkn---itdLDRSHFTFSSKVHIGFFHE 111  A. vaga
               hhhhhh-h   eeeeeee                eeeeeeee     eee  hhhhhhhhhh                    hhhhhh       5AGQ
                           β1                        β2      β3      α1                3_10      RBD
```

Supplementary Fig. 14. Alignment of DNA binding domains in MBD proteins.

Shown are the matches between *A. vaga* query proteins and the seed alignments in the cd00122 sequence cluster for (**a**) SETDB1-like proteins in cd01395 (HMT_MBD), (**b**) MeCP2-like proteins in cd01396 (MeCP2_MBD), and (**c**) BAZ2A-like proteins in cd01397 (HAT_MBD) in the hypertext format used by the NCBI Conserved Domains Database (CDD) (hyperlinked). Positions of DNA-binding residues are denoted by #; residues contacting with methyl groups are in boldface; secondary structure elements are denoted according to PDB entries at the bottom of each seed alignment.

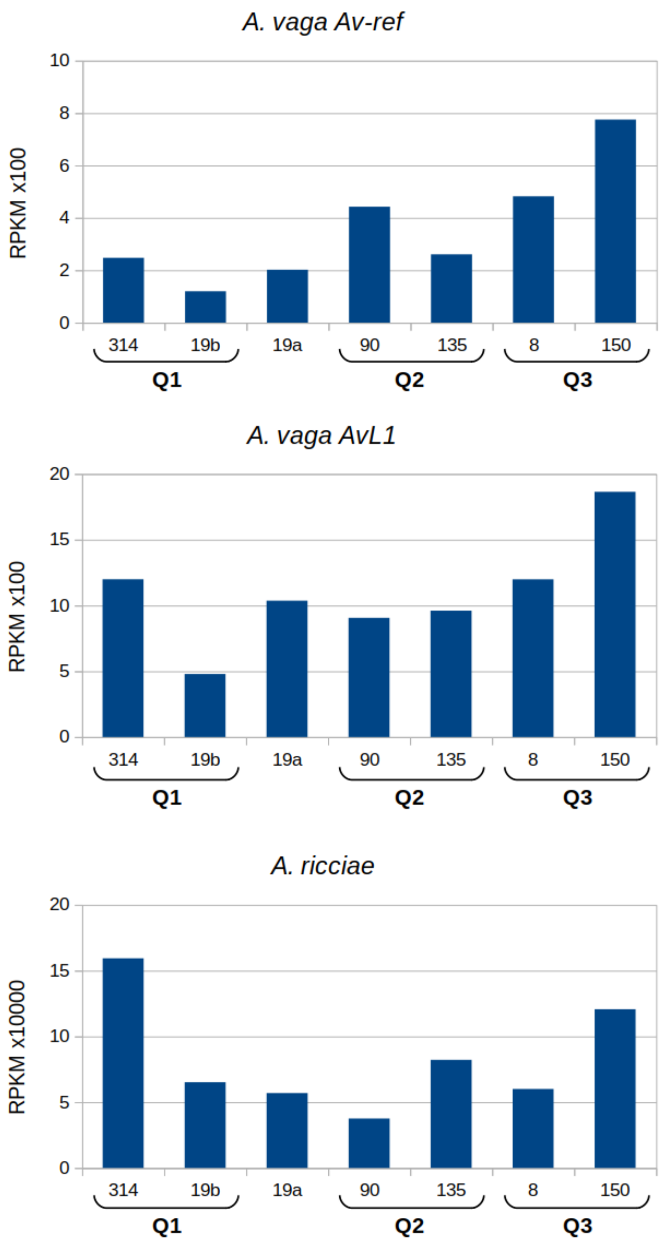

Supplementary Fig. 15. Transcription profiles of *Adineta* MBD-SETDB1-like genes.

Histograms show RNA profiles (RPKM) of (**a**) *Adineta vaga* reference (Av-ref), (**b**) *Adineta vaga* L1 strain and (**c**) *Adineta ricciae*. The initial Av-ref SETDB1 gene set (Supplementary Data 2) was used as queries in tblastn searches of AvL1 and *A. ricciae* assemblies (see Methods) to obtain the corresponding orthologs. After manual re-annotation, flanking regions were inspected for synteny to identify allelic and ohnologous pairs from the same quartet (Q). After annotation, RNA-seq counts were extracted using bedtools (multicov) and normalized with Reads Per Kilobase of transcript, per Million mapped reads (RPKM).

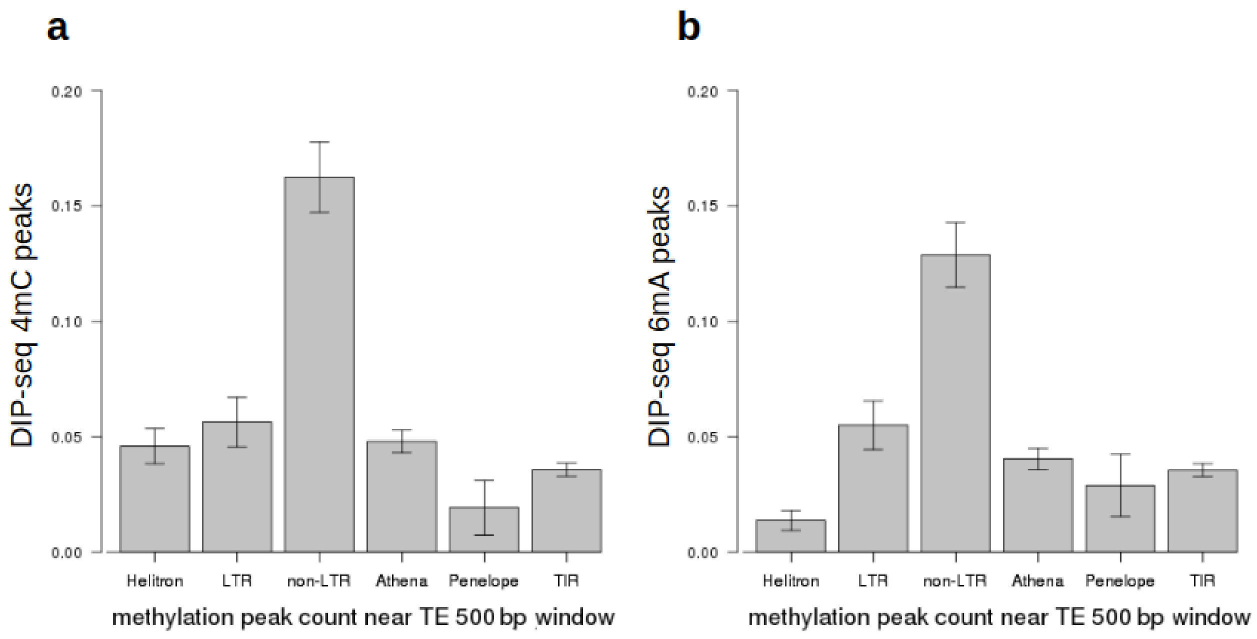

Supplementary Fig. 16. Distribution of 4mC and 6mA peak counts between different transposon types.

Shown are the mean peak counts (bar height) from DIP-seq data for 4mC (**a**) and 6mA (**b**) near each type of annotated TEs, classified as Helitron, LTR, non-LTR (LINE), Athena, Penelope or TIR, extending the 5' and 3' transposon ends by 500 bp window size. Error bars represent standard deviation for Helitron (n = 425), LTR (n = 315), non-LTR (n = 669), Athena (n = 522), Penelope (n = 161) and TIR (n = 4532). ANOVA analysis shows differences in distribution of 4mC (one-sided ANOVA, Df = 5, F = 39.871, p-val < 2.2 E-16) and 6mA peaks (one-sided ANOVA, Df = 5, F = 27.753, p-val < 2.2 E-16) near specific TE families. Crypton DNA TE copies (n = 13) are not included.

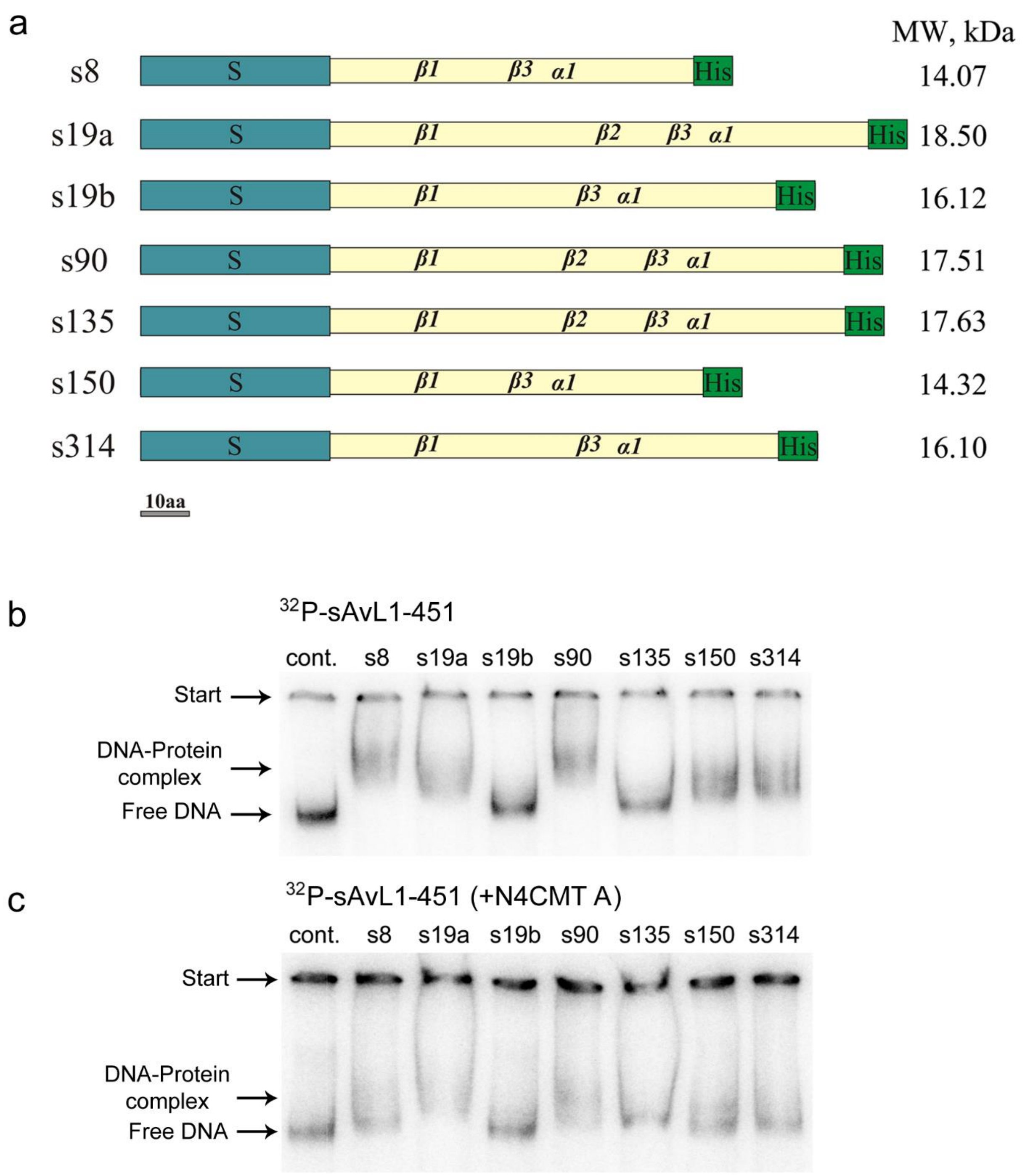

Supplementary Fig. 17. **MBD proteins from the *A. vaga* genome**.

a, Diagram of recombinant MBDs used in this study. S, S tag; His, His tag; MW, Molecular weight of protein in kDa. α1, β1, β2, β3, secondary structure elements outlined in Supplementary Fig. 14a.

b-c, Screening of recombinant AvMBD proteins for binding to unmethylated (**b**) and 4mC-methylated by N4CMT_A (**c**) DNA. For electrophoretic mobility shift assays, 2 ng of ^{32}P-sAvL1-451 were used with 50-100 ng of purified AvMBD proteins. Confirmation of the data from pilot screening was obtained in subsequent experiments, which are presented in Fig. 6 and Source Data 2.

SUPPLEMENTARY TABLES

Supplementary Table 1. Comparison of N6A methylation in *A. vaga* and other eukaryotes.

Phylum	Species	% 6mA/A	Symmetry	Motifs	Enzymes	Features	Transcripts	Methods	Reference
Rotifera	*Adineta vaga*	0.024	no	AGG,GAA	METTL4?	genes, TE	active	SMRT,IP	This study
Ciliates	*Tetrahymena thermophila*	0.4-0.8	yes (part)<br>ss, ds	AT<br>GATC	TAMT1<br>(MTA5)	Gene body,<br>linker+1+2	Activate,<br>weak corr.	SMRT	4<br>5
	Oxytricha nova	0.71-1.04		AT	MTA1	linker	mix up/dwn	MS,SMRT	6
Plants	Chlamydomonas	0.3-0.5		AT		TSS	Activate	IP,RE,exo	7
	Arabidopsis	.006-0.138	no	AGA,ACC		Genes, TE	gen ↑, TE ↓	MS,SMRT,IP	8
	Oryza sativa	0.2	no	GAGG	Ddm1<br>AlkB	Low at TSS<br>up at TTS	prom silent<br>body active	IP,MS,SMRT	9<br>10
Oomycetes	Phytophthora	0.04-0.05		AT	DAMT2[a]	TSS bi, TE	Lowly expr.	IP,MS	11
Fungi	Early-DF	0.2-2.8	yes	AT	PF02384[b]	genes	active	SMRT,IP,MS	12
	Dikarya	0.048-0.21	no	AV				SMRT	12
Ctenophora	Mnemiopsis	0.01-0.025	n/a		METTL4			ELISA	13
Ecdysozoa	*Caenorhabditis elegans*	0.01-0.4	no	AGAA<br>GAGG	DAMT1<br>AlkB			SMRT,MS,IP	14
	Drosophila	0.001-0.07	n/a		Tet/AlkB	TE	silence	MS,IP	15
	Aedes aegypti	0.00001	n/a		METTL4<br>Tet				16
	Bombyx mori		no	ACAA	METTL4	Low at TSS,<br>TTS	silence	IP-seq	17
Vertebrata[d]	Zebrafish	.002-.1 emb	no	AG		TE	activate	MS,IP	18
	Xenopus laevis	0.00009	no	AG		Low at TSS			19
	Sus scrofa	.05-.17 emb	n/a	-				MS	18
	Mouse ES<br>Brain<br>ESC	.0006-.007	no	 AAGA<br>AGGA	 <br>METTL4	 <br>noncoding	silence	 <br>MS,IP	20<br>21<br>22
	Rat	0.00001	n/a					MS,IP	23
	Human<br>Glioblastoma<br>2n/1n cells	.023-.064<br><br>0.004	no<br>H3K9me3	AGG<br><br>AG,GA	N6AMT1[c]<br><br>AlkBH1	exons, TTS<br>Low at X,Y<br>Allele-spec.	activate	 <br>SMRT	24<br>25<br>26

[a] Related to N6AMT2; [b] Related to MT type IC; [c] Related to N6AMT1; [d] 6mA presence in mammals is disputed[27].
Abbreviations: MS, mass-spectrometry; IP, MeDIP-seq; SMRT, SMRT-seq; TSS and TTS, transcription start and transcription termination sites; TE, transposable elements; emb, embryos; Early-DF, early-diverging fungi.

Supplementary Table 2. Putative amino-MTase and demethylase orthologs in the phylum Rotifera.

Species	N4CMT (N6_N4_Mtase) PF01555 SPPY	METTL4 (MT-A70) PF05063 DPPW	N6AMT1 PF05175 NPPY	N6AMT2 (N6aMIase) PF10237 DPPF/Y	MT type IC PF02384 NPPF/Y	AlkBH1	AlkBH4	TET	WGS assembly (source)
Adineta vaga (Av-ref)	+	+	+	+	-	+	+	-	[28]
Adineta vaga (AvL1)	+	+	+	+	-	+	+	-	[29]
Adineta ricciae	+	+	+	+	-	+	+	-	[30]
Adineta steineri	+	+	+	+	-	+	+	-	[31]
Rotaria magnacalcarata	+	+	+	+	-	+	+	-	[30]
Rotaria macrura	+	+	+	+	-	+	+	-	[30]
Rotaria sordida	+	+	+	+	-	+	+	-	[31]
Rotaria socialis	+	+	+	+	-	+	+	-	[31]
Rotaria sp. 'Silwood-1'	+	+	+	+	-	+	+	-	[31]
Rotaria sp. 'Silwood-2'	+	+	+	+	-	+	+	-	[31]
Didymodactylos carnosus	-	+	+	+	-	+	+	-	[31]
Brachionus plicatilis	-	-	+	+	-	+	+	-	[32]
Brachionus calyciflorus	-	-	+	+	-	+	+	-	[33]
Brachionus koreanus	-	-	+	+	-	+	+	-	[34]
Brachionus rotundiformis	-	-	+	+	-	+	+	-	[35]
Brachionus asplanchnoidis	-	-	+	+	-	+	+	-	[36]
Brachionus sp. 'Tiscar'	-	-	+	+	-	+	+	-	[36]

Supplementary Table 3. Properties of *E. coli* strains.

Strain name	Genotype	Methylation marks	Strain source
ER2925 (*dam-/dcm-*)	*ara-14 leuB6 fhuA31 lacY1 tsx78 glnV44 galK2 galT22 mcrA dcm-6 hisG4 R(zgb210::Tn10)*TetS *endA1 rspL136* (StrR) *dam13::Tn9* (CamR) *rfbD1 xylA-5 mtl-1 thi-1 mcrB1 hsdR2*	None (except rare *Eco*KI methylation)	NEB
ER2738 (methyl-free)	F´ *proA*$^{+}$*B*$^{+}$ *lacI*^{q} *Δ(lacZ)M15 zzf::Tn*10(Tet^{R})/ *fhuA*2 *glnV Δ(lac-proAB) thi-1 Δ(hsdS-mcrB)5*	None	NEB
M28	F⁻ *galK2(Oc) IN(rrnD-rrnE)1 rpsL200(strR) rph-1*	6mA, 5mC	M. Meselson
DH5α™	F- Φ80*lac*ZΔM15 Δ(*lac*ZYA-*arg*F) U169 *rec*A1 *end*A1 *hsd*R17(rk-, mk+) *pho*A *sup*E44 *thi*-1 *gyr*A96 *rel*A1 λ-	6mA, 5mC	Invitrogen
NEB® 5-alpha	*fhuA2 Δ(argF-lacZ)U169 phoA glnV44 Φ80Δ (lacZ)M15 gyrA96 recA1 relA1 endA1 thi-1 hsdR17*	6mA, 5mC	NEB
Top10	F- *mcrA* Δ(*mrr-hsd*RMS-*mcr*BC) Φ80*lac*ZΔM15 Δ*lac*X74 *rec*A1 *ara*D139 Δ(*ara,leu*)7697 *gal*U *gal*K *rps*L (StrR) *end*A1 *nup*G	6mA, 5mC	Invitrogen
BL21-AI™	F^{-}*omp*T *hsd*S$_{B}$ (r$_{B}^{-}$ m$_{B}^{-}$) *gal dcm ara*B::T7RNAP-*tet*A	6mA	Invitrogen
Rosetta™ 2(DE3)	F- *ompT hsd*SB(rB- mB-) *gal dcm* (DE3) pRARE2 (CamR)	6mA	Novagen

Supplementary Table 4. Genometric correlations between DIP-seq methylation marks and TE annotations in Av-ref and AvL1 assemblies [37].

Genometric Correlation	Assembly	Av-ref		AvL1	
IP-seq to TE annotations	Test	IP 4mC-TEs	IP 6mA-TEs	IP 4mC-TEs	IP 6mA-TEs
Relative Ks p−value[a]	Kolmogorov-Smirnov test	1.2897e−13	0.0357944	0.00833478	0.00483626
Relative ecdf deviation area[b]	Permutation test	0.0459	0.0070	0.0125	0.0137
Relative ecdf area correlation[b]		0.1842	0.0285	0.0502	0.0540
Relative ecdf deviation area p−value[b]		<0.01	0.01	<0.01	<0.01
Jaccard Measure p−value[c]	Jaccard test	<0.01	<0.01	<0.01	<0.01
Jaccard Measure lower tail[c]		FALSE	FALSE	FALSE	FALSE
Projection test p−value[d]	Projection test	0	0	0	0
Projection test lower tail[d]		FALSE	FALSE	FALSE	FALSE

[a]Relative Ks (Kolmogorov-Smirnov one-sided test) p−value: relative distance test measures whether two sets of positions are closer together or further apart than expected. P-value close to zero: non-uniform distribution (query locations are non-independent of the references).
[b]Relative ecdf deviation area p−value: compares the two cumulative distribution functions using the area of the region in which they differ as the test statistic. We performed a permutation test (two-sided) for significance. P-value close to zero: query features are closer than expected to the reference features.
[c]Jaccard Measure lower tail: measures overlaps between two interval sets by measuring the extent of intersection between two interval sets, divided by the length of their union. FALSE: Overlap is more frequent than expected (two-sided Jaccard test p-val <0.01).
[d]Projection test lower tail: query intervals are represented as midpoints, but the reference should be a set of intervals. Features are closer to (TRUE) or away from the reference (FALSE, two-sided Projection test p-val <0.01).

Supplementary Table 5. Genome assembly and gene annotation metrics.

Species	*A. vaga* Av-ref [a]	*A. vaga* AvL1	
Accession (assembly name)	GCA_000513175.1 ('2013')	AvL1 initial assembly[b]	AvL1 hybrid assembly[c]
Coverage[d] (mean)	Not calculated	40.74 (Illumina), 29.82 (PacBio)	33.88 (Illumina), 33.33 (PacBio)
Span (Mb)	217.9	197.1	217.1
No. contigs	36,335	19,202	9,859
Contig N50 (kb)	96.7	22.1	87.4
No. scaffolds	36,167	19,202	9,859
Scaffold N50 (kb)	260.3	19.2	87.4
Scaffold N90 (kb)	7.8	5.3	15.8
Scaffold longest (kb)	1,087.3	167368	747.8
Gaps (Ns) span (kb) (% genome)	4,136.3 (1.9%)	None	None
% GC	30.8	29.9	30.4
BUSCO EUK (n = 429)	C: 89%; F: 3%; D: 8%	C: 84%; F: 8%; D: 8%	C: 88%; F: 6%; D: 6%
BUSCO MET (n = 843)	C: 84%; F: 3%; D: 13%	C: 79%; F: 6%; D: 15%	C: 81%; F: 5%; D: 14%
Annotation			
Method	Augustus	Augustus/GeneMark.ES	Braker/Augustus
No. genes	49,300	61,531	65,934

[a]*A. vaga* assembly CGA_00513175.1 [28].
[b]AvL1 initial assembly (no contaminants) GCA_013411005.1 [29].
[c]AvL1 curated hybrid assembly (Illumina + PacBio) GCA_021403095.1 from this study.
[d]Average read coverage based on trimmed and filtered data.
Abbreviations: GC, guanine-cytosine; BUSCO, Benchmarking Universal Single-Copy Orthologs; EUK, Eukaryota; MET, Metazoa; C, Complete; F, Fragmented; D, Duplicated.

Supplementary Table 6. SMRT-seq base modification detection.

PacBio base modification: total nucleotides		4mC		6mA	
bases	total N's Assembly	4mC-10x[a]	4mC-20x[b]	6mA-10x[c]	6mA-20x[d]
	32676087 C's + 75836765 A's	21016	10369	17886	6926
PacBio base modification: total bins with modification					
bins[e]	total bins	4mC-10x	4mC-20x	6mA-10x	6mA-20x
1kb	221401	15965	6536	15078	5364
5kb	49278	11611	4473	11371	3918
10Kb	28432	9001	3503	8937	3131
Bins-TEs[f]	total bins	4mC-10x	4mC-20x	6mA-10x	6mA-20x
1kb	13552	679	235	601	204
5kb	9973	1577	505	1471	420
10Kb	9542	2326	694	2262	604
Bins-Genes[g]	total bins	4mC-10x	4mC-20x	6mA-10x	6mA-20x
1kb	181469	15118	6270	14144	5036
5kb	88412	26892	10518	26346	9161
10Kb	76640	35659	14197	35209	12537

[a]SMRT-seq 4mC modified bases with minimum 10x PacBio read coverage
[b]SMRT-seq 4mC modified bases with minimum 20x PacBio read coverage
[c]SMRT-seq 6mA modified bases with minimum 10x PacBio read coverage
[d]SMRT-seq 6mA modified bases with minimum 20x PacBio read coverage
[e]Genome binning into different bin sizes (1, 5 and 10 kb)
[f]Bins containing TE annotations
[g]Bins containing gene annotations

Supplementary Table 7. Primers and oligonucleotides.

Name	Sequence (5'->3')	Purpose
N4CMT-F<br>N4CMT-R<br>N4CMT-Cbx-R	tttGGATCCgtcatttactaacaaatatgtcgtt<br>tttCTCGAGccacaaatgtacttttgacttcgat<br>tttCTCGAGttgtctKMgacgtaatcgataacca	*N4CMT* ORF amplification
N4CMT_Seq1	atcgcggttcgacagtcaat	Sequencing
IAY21-SP-F<br>IAY21-SP-R<br>IAY21-OP-F<br>IAY21-OP-R<br>IAY22-OP-F<br>IAY21-Y65A-F	aattcgaacgccagcacatg<br>gatctcagtggtggtggtgg<br>ccagccaataaacttggtcttcgtgaaggt<br>tggagctgtaacaacacattgaacgga<br>ccagccaataaacttggccttcgtga<br>ttgttacagctccaccagc	Site-directed mutagenesis to obtain catalytic mutants
		Substrates for in vitro assays:
MTase_subst-1a<br>MTase_subst-1b	ttgaatagttccgCCGgaattttCagtcaa<br>ttgactGaaaattcCGGcggaactattcaa	4mC 30-bp from *A. vaga* AvL1 c1882
Av11_tel_GGGTGTGT<br>Av11_tel_CCCACACA	gggtgtgtgggtgtgtggg<br>cccacacacccacacaccc	*A. vaga* AvL1 19-bp telomeric-like repeat
Av_tel_TGTGGG<br>Av_tel_ACACCC	tgtgggtgtgggtgtggg<br>acacccacacccacaccc	*A. vaga* Av-ref 18-bp telomeric repeat
Av11_c2350-F1<br>Av11_c2350-R1	agggagacatccttattgaagca<br>tcaagtctgttgacctacataagaa	4mC ~460-bp repeat unit from *A. vaga* AvL1 c1882
Av11_4mC/6mA_F1<br>Av11_4mC/6mA_R1	tgtaccctgcagatgttttgtg<br>tctcagacgggctacatgat	4mC/6mA 580-bp from AvL1 c785 (*Athena* retroelement)
Av11_6mA_F1<br>Av11_6mA_R1	tccgcccattccataactgt<br>catcatgttgtcaaaggaaatcc	6mA, 405-bp from *A. vaga* AvL1 c699 (hnRNP-A)
A11motif-HindIII-F<br>A11motif-BamHI-R	tttAAGGTTacctcaatgcacatatagcc<br>tttGGATCCtttgatatgcttcaataaggatg	Contains putative N4CMT recognition motif from *A. vaga* AvL1 c1882 repeat
A11motif-3'209-F1	tcaCcctaccctcatggatt	4mC 209-bp part of repeat unit from *A. vaga* L1 c2350. Used with Av11_c1882-R1 primer.
A11motif-5'200-R1	aatcgatgcttggtttgagc	4mC 200-bp part of repeat unit from *A. vaga* L1 c1882. Used with Av11_c2350-F1 primer.
AvL1c2350-364-R	ggatctatgttgagtgtgtgg	4mC 371-bp part of repeat unit from *A. vaga* L1 c1882. Used with Av11_c2350-F1 primer.

Supplementary Table 8. N4CMT recombinant proteins.

Protein_ID	Protein_variant	Length, aa	MW, kDa	pI
N4CMT A’	*N6_N4_MTase*+Cbx (s23)	426	49.73	8.98
N4CMT A	*N6_N4_MTase*+Cbx (s23) p.L416R	426	49.78	9.05
N4CMT B	*N6_N4_MTase*+Cbx (s179)	426	49.75	9.06
N4CMT B’	*N6_N4_MTase*+Cbx (s179) p.R416L	426	49.71	8.99
N4CMT A-ΔCbx	*N6_N4_MTase* (s23)	287	33.20	8.60
N4CMT B-ΔCbx	*N6_N4_MTase* (s179)	287	33.13	8.60
N4CMT A-APPA	*N6_N4_MTase*+Cbx (s23) p.S62A;Y65A;L416R	426	49.63	8.99
N4CMT B-APPA	*N6_N4_MTase*+Cbx (s179) p.S62A;Y65A	426	49.65	9.07

Supplementary Table 9. Summary of N4CMT action on *E. coli* genomic DNA in vitro.

E. coli strain	Acquisition of 4mC mark after N4CMT treatment	*E. coli* genetic background	
		6mA	5mC
Rosetta 2(DE3) (n=2)	++	Dam+ / EcoK1+	Dcm-
BL21-AI	++	Dam+ / EcoK1+	Dcm-
M28	+	Dam+ / EcoK1+	Dcm+
ER2925 (n=2)	+	Dam- / EcoK1+	Dcm-
ER2738	-	Dam- / EcoK1-	Dcm-

Supplementary Table 10. Summary of ChIP-seq peaks identified by MACS2 (diagonal values) and overlap of peaks within Av-ref and AvL1 assemblies.

Av-ref	H3K4me3	H3k9me3	H3K27me3
H3K4me3	5163	13	27
H3K9me3	-	1902	1811
H3K27me3	-	-	4630
AvL1	H3K4me3	H3k9me3	H3K27me3
H3K4me3	5789	43	48
H3K9me3	-	1205	681
H3K27me3	-	-	2378

Supplementary Table 11. Methylation analysis in under-annotated regions.

contig	start	stop	4mC-10x	6mA-10x	annotations
Contig1073[a]	0	25272	10	17	Chapaev
Contig1204[e]	66205	74905	4	10	
Contig1251[a]	29664	39261	11	10	Polinton-9
Contig126[b]	34974	38796	2	10	Hebe
Contig1397[b]	24097	31635	9	10	Juno/AthenaJN
Contig1606[e]	50662	52438	10	5	
Contig1615[e]	4175	10552	9	12	
Contig1743[e]	0	5188	10	10	
Contig18953[e]	26550	30514	7	14	
Contig2220[a]	29384	35415	10	6	Ginger
Contig2425[b]	43254	59525	1	11	CACTA1
Contig2467[b]	0	19427	13	2	Helitron
Contig286[c]	0	7155	1	12	ITS
Contig2879[e]	2234	4406	19	17	
Contig2948[a]	0	8375	7	10	Helitron
Contig3423[e]	1154	6396	13	16	
Contig3571[d]	0	10942	24	14	TR
Contig3784[e]	0	9651	8	20	
Contig3893[b]	4644	35659	14	4	Athena-P
Contig4128[c]	0	5620	24	16	ITS
Contig4313[a]	8885	19081	10	4	DNA-N26B
Contig4665[e]	840	5755	34	10	
Contig480[b]	0	13847	11	5	Athena-I
Contig523[e]	36990	42099	12	5	
Contig5325[b]	8714	25663	15	22	Athena-M
Contig6065[b]	3940	10974	22	18	TelKA1a
Contig61067[b]	0	10836	12	18	Vesta1
Contig67[e]	0	10315	11	10	
Contig805[e]	3363	6455	3	13	
Contig8145[e]	15264	20597	4	11	
Contig839[e]	38934	43045	11	5	
Contig865[e]	0	11998	11	13	
Contig882[b]	6424	20046	11	4	MuDR/Mariner
Contig89[b]	14890	25375	7	11	Vesta1
Contig925[e]	105036	112889	13	9	
Contig991[b]	2840	24886	10	2	TR/Sola3/Pen

[a]Repbase-TE (5); [b]Adineta-TE (12); [c]ITS (2); [d]tandem repeat (1); [e]unknown (16).

SUPPLEMENTARY NOTES

Supplementary Note 1: Genometric correlation analysis of DIP-seq datasets

To find out whether peaks are distributed non-randomly, we examined the statistical significance of genomic correlations between peak distribution and annotated genomic features. Since functional interactions often depend on spatial proximity between the reference feature and the density of query features relative to it, we used spatial correlations as a proxy for functional analysis [37] (Supplementary Table 4, Supplementary Fig. 4). Genometric correlation analysis on annotated Av-ref scaffolds shows that 4mC peaks and TEs are non-uniformly distributed (p-value: 1.29E-13, Kolmogorov-Smirnov test), and that the query features (4mC peaks) are closer than expected to the reference features (TEs) (Jaccard and permutation test). In contrast, we find that 6mA peaks and TEs are more uniformly (randomly) distributed (p-value: 0.036, Kolmogorov-Smirnov test), and that 6mA peaks tend to be further away from TEs (permutation test). When gene annotations are used as reference points, both 4mC and 6mA modifications are uniformly distributed, but for 6mA peaks the distance from genes is consistently small, while for 4mC peaks the distance from genes tends to be larger (Jaccard and permutation test).

The distribution of 4mC and 6mA DIP-seq was similarly correlated in the AvL1 strain for its annotations (genes and TEs). Genometric correlation analysis on AvL1 showed that both modification peaks, 4mC and 6mA, have a small absolute positive correlation (p-value: 0.0083 and 0.0048, Kolmogorov-Smirnov test, respectively) (Supplementary Table 4, Supplementary Fig. 4) and are also closer than expected to TEs as reference features than to gene models (Jaccard and permutation test). Overall, correlation analyses on DIP-seq data in both Av-ref and Av-L1 suggest that DIP-seq 4mC marks are not uniformly distributed and have a preferential localization near TEs in both genomes.

Supplementary Note 2: In vitro activity and substrate specificity of N4CMT

We sought to find out whether recombinant N4CMT displays the expected in vitro activity, to evaluate its preference for different substrates. To this end, N4CMT was expressed in *E. coli*, partially purified by immobilized metal-affinity chromatography (Supplementary Fig. 9b,c), and used to methylate *E. coli* gDNA in 1x *M.Bam*HI buffer (NEB) supplemented with S-adenosylmethionine as a donor of methyl groups (Methods). Further, to check if pre-existing N6A- and C5-methyl groups could modulate the efficiency of N4C methylation, we used as substrates gDNA from five *E. coli* strains differing by genetic backgrounds with regard to methylation: Rosetta 2(DE3) and BL21(DE3) (both *dam+ dcm-*), derived from *E. coli* B, and three *E. coli* K12 derivatives (methyl-positive M28 (*dam+ dcm+*) and methyl-negative ER2738 (*dam- dcm- EcoK1-*) and ER2925 (*dam- dcm-*)) (Supplementary Table 3). After incubation, samples and control DNAs were spotted on two identical membranes and probed with anti-4mC and anti-6mA antibodies, respectively; the latter served as an internal control and agreed with expectations from the genetic background of each strain (Fig. 3d; Supplementary Fig. 9h). Interestingly, *dam+ dcm- E. coli* B derivatives displayed stronger signal than *dam- dcm-* and *dam+ dcm+* strains, suggesting that pre-existing 6mA marks might facilitate 4mC addition, and that the presence of 5mC in the carbon ring of the cytosine may interfere with 4mC addition at the neighboring amino group. Activity in *E. coli* strains is summarized in Supplementary Table 9.

We also checked N4CMT for in vitro activity on dsDNA substrates (Supplementary Fig. 9f). The positive control (*M.Bam*HI-methylated pUC19) was readily detected with anti-N4mC antibodies. However, unmethylated pUC19 and pBluescript SK+, grown in the *dam- dcm-* background, acquired only barely detectable 4mC marks upon N4CMT treatment. A more favorable in vitro substrate was *N4CMT* itself, as inspection of AvL1 PacBio data revealed 4mC marks over its ORF, indicating that it serves as its own substrate in vivo. Indeed, PCR-amplified *N4CMT_A* and *B* fragments yielded 4mC signal in vitro, hinting at the possibility of self-regulation in vivo.

The inability of empty plasmids to serve as efficient substrates may be explained by the lack of cytosines in a rotifer sequence context favored by N4CMT. We performed in vitro assays on the ~460-bp tandem repeat from AvL1 DNA with high density of DNA modifications (see above; Supplementary Fig. 9d,e), reasoning that it would serve as an efficient substrate in vitro. The PCR primers, spanning 451 bp of the repeat, amplified 1, 2 and 3 repeat units, which were separately used as substrates. We also annealed two complementary oligonucleotides to form a 30-bp dsDNA fragment containing the cytosines modified with 100% efficiency in SMRT-seq data (Supplementary Fig. 9f; Supplementary Table 7). The 451-bp fragment indeed served as an efficient substrate in vitro (Supplementary Fig. 9f,g), although increasing the number of repeat units did not improve methylation efficiency (Supplementary Fig. 9i). However, the short 30-bp fragment failed to yield detectable signal, even when used in large amounts (Supplementary Fig. 9f). Another short G-rich substrate, made of annealed GT-rich repeat-containing complementary oligonucleotides (Supplementary Table 7), also

failed to acquire 4mC (not shown). In agreement with in vivo results, the SPPY→APPA catalytic mutants were unable to add 4mC to the 451-bp fragment, reconfirming the identity of catalytic residues in vitro and ruling out any co-purifying MTases (Fig. 3e). To test for 6mA addition, we used PCR-generated 405- and 589-bp fragments marked by 6mA in AvL1 SMRT-seq data (Supplementary Table 7). No 6mA was acquired upon incubation with N4CMT, suggesting that it lacks 6mA MTase activity (not shown).

We further dissected the 451-bp fragment into sub-fragments of 127 bp and 357 bp, to check whether they would serve as substrates (Supplementary Fig. 9e). While the 357-bp fragment was readily modified by N4CMT, the 127-bp fragment was not, perhaps due to minimal length requirements (Supplementary Fig. 9h,i). Alternatively, it may be that N4CMT, which underwent horizontal transfer from a prokaryote relatively recently on an evolutionary time scale in comparison with METTL4- or DNMT-like MTases, retains target specificity in its TRD and prefers one fragment over the other. In support of this idea, we identified a partially homologous 750-bp AvL1 tandem repeat, similarly associated with an *Athena* retroelement. Aligning it with a 481-bp tandem repeat from another AvL1 contig with high density of modifications, we defined a bipartite motif common to these repeat units (Supplementary Fig. 9d,e). We searched Av-ref and the sibling species *A. ricciae* for sequences homologous to the AvL1 460-bp repeat. In Av-ref, we identified 5 contigs with 174-, 482- and 660-bp tandem repeat units partially homologous to AvL1 repeat, all adjacent to *Athena-W1*; in *A. ricciae,* two contigs carried 6 and 11 units of a 490-bp tandem repeat 74% identical to AvL1, with the bipartite motif (Supplementary Fig. 9j).

Dividing the 451-bp fragment into two approximately equal parts (200 and 209 bp) yielded no detectable signals (Supplementary Fig. 9g), possibly due to insufficient fragment size. Since the positive sAvL1-357 bp fragment contains only one part of the bipartite motif, we tested a similarly sized PCR fragment (sAvL1-371 on Supplementary Fig. 9e) containing the other part of the motif. However, it was not methylated by N4CMT, suggesting that this part is not essential in vitro (Supplementary Fig. 9h), although it may play a role in vivo. Most importantly, insertion of short 97-bp or 119-bp fragments with the bipartite motif (Supplementary Fig. 9d,e) into pUC19, initially unable to act as a substrate, converted it into an efficient in vitro substrate (Supplementary Fig. 9g). Thus, an MTase of bacterial origin shows preference for certain recognition sequences, which might have served as its targets in the distant evolutionary past.

Supplementary Note 3: Gene transcription and DNA modifications

According to SMRT-seq, repetitive regions such as TEs or TRs attract the highest base modification density (Fig. 2g). Still, nearly one-half of tag counts originate from genic loci: a total of 10,928 4mC and 9,596 6mA methylation marks, representing *ca.* 52% and 54% of total 4mC and 6mA, respectively, lie within gene annotations. To examine the links between gene transcription and DNA methylation at base-level resolution, we compared AvL1 transcriptomic data for genes carrying one or more methylation marks in the SMRT-seq dataset (Supplementary Fig. 11a-c), distinguishing those with 1, 2, 3 or more marks (with 4mC and 6mA separately and combined). We used RPKM values to divide genes into subsets with higher (RPKM≥1) and lower (RPKM<1) transcription levels, and to examine correlations with the number of methylation marks. In general, numbers of genes with methylated sites (4mC, 6mA or both combined) and higher RPKM (≥1) were significantly higher than those with equal methylation levels but lower RPKM (<1). Further, although genes with >3 6mA sites did not show significant differences (p-val = 0.71, χ^2 test for 40 and 33 genes for high and low RPKM respectively), the combined numbers for 4mC and 6mA were significant (p-val = 3.02e-5) (Supplementary Fig. 11c). Even though some of the methyl marks may be false positives, the observed difference between two gene categories suggested association between methyl marks and genes with higher transcription levels.

To uncover connections between gene methylation and transcription, we explored the occupancy of 4mC and 6mA near the TSS in genes. Regardless of the number of modified bases in gene body or in a 2-kb window upstream of TSS, methylated genes are consistently expressed at higher levels than unmethylated genes (Supplementary Fig. 11d). Notably, the 6mA occupancy shows a characteristic profile, i.e. a double peak, upstream of TSS, while 4mC does not (Supplementary Fig. 11e, right panel). Inspection of these patterns with cluster analysis (deepTools2 option --kmeans with --outFileSortedRegions) shows that they mainly originate from 1212 gene models carrying the double 6mA mark (*ca.* 260 bp and 750 bp upstream of TSS) and no significant accumulation of 4mC sites (Supplementary Fig. 11f). The increased 6mA deposition was corroborated by DIP-seq data, with a significant peak observed upstream of TSS for these 1212 genes (Supplementary Fig. 11g); a smaller 4mC DIP-seq peak was also visible further upstream. Comparison of expression levels for these 1212 genes shows that their transcription levels are higher than average (Supplementary Fig. 11h). We then checked their Av-ref homologs for similarity of methylation and expression profiles. After blastp search with 1212 AvL1 genes as queries, we obtained 929 *A. vaga* homolog gene models, which not only show a similar DIP-seq peak profile (Supplementary Fig. 11i), but also had significantly higher than average expression levels (Supplementary Fig. 11j). Overall, SMRT-seq modification data agree well with DIP-seq profiles of homologous gene sets in two strains. These observations minimize the possibility of RNA-derived m^6A signal and support the view that genic 6mA modifications, particularly near the TSS, are positively correlated with higher expression levels of the corresponding genes.

We also performed gene ontology (GO) analysis to find out whether specific gene categories are subject to modification. In AvL1, 66% of genes with 6mA SMRT-seq signature at TSS had annotated functions, which is comparable with 63% for all gene models (41,340 genes with GO annotations out of 65,934) (Source Data 1). Several GO categories showed significant differences between 6mA-methylated and unmethylated genes (two-sided Fisher's exact test), and the groups shared between Av-ref and AvL1 methylated genes were broad (metabolism, development, catalytic activity, biogenesis). Results were visualized in scatterplots generated with REVIGO [38] (Supplementary Fig. 12). These findings are consistent with designation of 6mA as a developmentally dynamic mark [15,19,39].

Haplotype-specific 6mA patterns were suggested to affect allele-specific transcription [26]. Since the AvL1 genome displays the same degenerate tetraploid structure as Av-ref, with 40% of the genome organized in quartets [28,29], we searched for allele-specific DNA methylation patterns affecting homologs and/or ohnologs (homeologs). We defined collinear block regions in AvL1 (see Methods) and searched for inter-block differences in base modifications (SMRT-seq) and in transcription levels (log_2RPKM). Initial inspection suggested that any inter-block transcription differences (Supplementary Fig. 10b) originated from blocks with broken collinearity (i.e., when blocks could not be aligned without rearrangements). Upon comparing the number of base modifications between homologous blocks, only a few cases showed disparity in modified bases (Supplementary Fig. 10c). Out of 28705 pairs in AvL1, with 25619 defined as collinear and 3086 with broken collinearity, 615 and 59 show difference in SMRT-seq methylation of two or more marks (square root of base modification difference) for collinear and broken pairs, respectively. To establish if collinearity (collinear or broken) and difference in methylation between blocks are independent, $\chi 2$ test was performed using the categories of pairs without base modification difference (value 0 for 16000 collinear pairs vs. 1743 broken pairs) and pairs with any difference (value ≥ 1 in 17611 collinear pairs vs. 1343 broken pairs). The test showed that proportions between both categories are not fully independent, with some association between collinearity and methylation level difference between pairs ($\chi 2$ test, p-val = 3.57E-21).

Finally, we analyzed the remaining AvL1 genomic regions lacking *ab initio* annotations but still displaying significant methylation density. Genomic regions without gene models or annotated TEs/TRs were extracted, and each region was examined for the presence of 4mC and/or 6mA detected by SMRT analysis. Contigs with methyl marks were inspected for coverage with Illumina, PacBio and RNA-seq reads, and showed a lack of transcriptomic coverage, indicating transcriptionally silent regions (Supplementary Fig. 7f; Supplementary Table 11). Regions covered by methyl marks were extracted and used in BLAST searches to detect homology to known genes or TEs. From a final set of 36 AvL1 loci with significant numbers of 4mC and/or 6mA sites (>10 tags), the analysis revealed one under-annotated tandem repeat, two ITS regions and 17 regions with homology to TEs (12 from combined *Adineta* TE libraries and 5 from Repbase), indicating that one-half of the extensively methylated, transcriptionally silenced regions represents under-annotated TEs.

Supplementary Note 4: Methylomes and small RNAs

The exceptionally low TE content and diversified small RNA (sRNA) silencing machinery in bdelloids, averaging 20 Piwi/Ago and 30 RdRP variants, implies tight controls on TE proliferation via efficient silencing [28,31]. In *A. vaga*, virtually every active TE family displays coverage by pi-like RNAs, which is correlated with low transcriptional activity [40]. Indeed, we find that the significant overlap between TEs and heterochromatin, as defined by H3K9me3 and H3K27me3 depositions shown in Fig. 4b,d, is paralleled by an overlap between TEs and sRNAs aligned to Av-ref (Supplementary Fig. 13a; Methods). We thus analyzed sRNA association with histone marks and with 4mC/6mA DIP-seq peaks. Relative fold excess of sRNA is evident at heterochromatic H3K9me3 and H3K27me3 peaks and extends into nearby regions; in contrast, sRNA enrichment is low within H3K4me3 peaks, which mark active genes (Supplementary Fig. 13b). Comparison of sRNA *vs* DIP-seq peaks for 4mC/6mA shows enrichment within DIP-seq peaks, with 4mC peaks having higher relative fold enrichment than 6mA (Supplementary Fig. 13c). To estimate the proportion of DIP-seq peaks contributing to each sRNA profile, we clusterized the peaks with the k-means algorithm, sorting by sRNA coverage (deepTools2 option --kmeans --outFileSortedRegions), which showed that ~25% and ~15% of 4mC and 6mA peaks, respectively, display small RNA enrichment. The overlap between sRNA and DIP-seq peaks is localized to the peak area, while sRNA enrichment at heterochromatic ChIP-seq peaks extends further into adjacent sequences, which may indicate spreading. Although the exact pathways linking piRNAs to histone and DNA methylation layers remain to be defined, the highly diversified set of PIWI proteins may serve as connectors to both epigenetic layers.

SUPPLEMENTARY DISCUSSION

Base modification, primarily in the form of methylation, constitutes an important facet of epigenetics due to the covalent nature of its linkage to DNA. In eukaryotes, the archetypal 5mC modification dominates the epigenetic landscape, and its distribution patterns are established by concerted action of writers, readers and erasers of epigenetic marks. Eukaryotic maintenance and *de novo* MTases *Dnmt1* and *Dnmt3* act together with demethylases to set the levels of CpG methylation [41,42], and may have been doing so since the divergence of plants and animals [43] (but see [44]). In bacteria, the most widespread DNA modifications added by amino-MTases of R-M systems modify the exocyclic amino groups of adenines and cytosines, with 5mC constituting a distant third [45] because of high incidence of 5mC→T transitions prone to deamination. The 6mA modification is widespread in eukaryotes, although its role is still controversial, especially when its levels are close to detection limits [46-49], and may eventually be settled only upon unambiguous identification of the underlying enzymatic machinery. Our initial assessment of the *A. vaga* methylome broadly agrees with the view of 6mA as a dynamic context-dependent mark, which is associated with higher expression in a subset of genes but is also found over repressed TEs.

The overall level of 4mC in *A. vaga*, as revealed by SMRT-seq, amounted to 0.065% of cytosines, while the somewhat lower 6mA content (0.024% of adenines) is much higher than the reported 6mA levels in *X. laevis* or mouse, which are close to background values [27], and is comparable to values reported for *C. elegans*, *Drosophila*, and plants (Supplementary Table 1). Lower 6mA levels are not surprising, as this mark is dynamic and can be modulated in a tissue- and stage-specific fashion by balancing activities of N6A-MTases and AlkB-like demethylases, with several candidates present in bdelloids. Indeed, a higher fraction of 4mC may be due to the lack of enzymes responsible for active cytosine demethylation, such as TET or potential homologs of bacterial R-M enzymes recognizing 4mC (Supplementary Table 2). Notably, both 4mC and 6mA sites show an asymmetric pattern, in contrast to symmetrical MTases, such as Dnmt1 in mammals or N6A-MTases of ciliates and early-diverging fungi, which act on hemi-methylated DNA [6,12,50]. The lack of maintenance MTases acting on symmetric motifs implies that methyl marks should be added *de novo* after DNA replication in order to be maintained at specific sites.

Interestingly, the preferred symmetrical dinucleotide for 4mC addition (CpG) coincides with that of canonical C5-MTases, although the asymmetric CpA is also frequently utilized. This agrees with higher similarity of N4CMT to bacterial MTases with a CG doublet in their recognition motif, suggesting that recognition by TRD may contribute to target choices. For 6mA addition, the asymmetric ApG or GpA are the preferred sites, as in other metazoans with similar 6mA content

(Supplementary Table 1). Symmetrical 6mA addition to ApT dinucleotides occurs in green algae, ciliates, and early-diverging fungi, where it is associated with actively transcribed genes and with linker DNA between nucleosomes [4,6,7,12]. The sequence context of methylation sites may further contribute to recognition of methyl marks by various readers, often resulting in opposite transcriptional effects, as shown for 5mC or 6mA [22].

It is hardly a coincidence that N4CMT is most closely related to MTases of cyanophages rather than bacteria. Indeed, phage-borne orphan MTases, in addition to being prone to horizontal spread, may be under evolutionary pressure to broaden their sequence specificity to protect the phage from multiple bacterial R-M systems [51]. An MTase with strict target specificity is unlikely to cover a broad range of epigenetic targets, limiting its regulatory potential, and would benefit from reduced sequence specificity while acquiring chromatin-based targeting. The intrinsic N4CMT target preference adds an intriguing twist to this view. While this preference is seen in our in vitro assays and is manifested in vivo as high-density SMRT modification regions, these regions do not show an increased density of H3K9/27me3 histone marks over modified TRs (Supplementary Fig. 7a). In *C. elegans*, a SETDB1 homolog met-2 adds H3K9me2 marks to suppress transcription of satellite repeats, which in *met-2* null worms yield DNA-RNA hybrids and trigger DNA damage-induced germline lethality [52]. Although investigations of DNA damage are outside the scope of the present work, future studies may uncover additional pathways involving chromodomain-independent N4CMT activity targeting 4mC to silence satellite repeats. Indeed, the 460-bp repeat in AvL1 is fully silenced, as our qRT-PCR experiments failed to yield PCR products. It is possible that N4CMT was initially recruited by the bdelloid ancestor through its ability to recognize specific sequence motifs, and entered the epigenetic system only later, upon acquisition of the chromodomain and evolution of 4mC preference by a subset of SETDB1 paralogs.

It also remains to be seen whether H3K9me and H3K27me exhibit spatial and functional overlap in bdelloids, as in ciliates [53,54], or are separated in space/time, with such studies being impeded by syncytial organization. Although in *Drosophila* and mammals H3K9 denotes constitutive and H3K27 - facultative heterochromatin, and the relevant enzymatic machinery is represented by SUV39H, G9a and SETDB1 homologs for H3K9 and the EZH-containing Polycomb repressive complex 2 for H3K27, the lack of SUV39H-like and G9a-like proteins in bdelloids may indicate their replacement with diversified SETDB1 paralogs for H3K9 methylation, and supports a facultative, bivalent nature of their heterochromatin, as indicated by triple Tudor domains in SETDB1 which recognize a combination of active and repressive histone marks to ensure silencing [55].

SUPPLEMENTARY REFERENCES

1. Sullivan, M.J., Petty, N.K. & Beatson, S.A. Easyfig: a genome comparison visualizer. *Bioinformatics* **27**, 1009-1010 (2011).
2. West, P.T., Probst, A.J., Grigoriev, I.V., Thomas, B.C. & Banfield, J.F. Genome-reconstruction for eukaryotes from complex natural microbial communities. *Genome Res* **28**, 569-580 (2018).
3. Jumper, J. *et al.* Highly accurate protein structure prediction with AlphaFold. *Nature* **596**, 583-589 (2021).
4. Wang, Y., Chen, X., Sheng, Y., Liu, Y. & Gao, S. N6-adenine DNA methylation is associated with the linker DNA of H2A.Z-containing well-positioned nucleosomes in Pol II-transcribed genes in Tetrahymena. *Nucleic Acids Res* **45**, 11594-606 (2017).
5. Luo, G.Z. *et al.* N(6)-methyldeoxyadenosine directs nucleosome positioning in Tetrahymena DNA. *Genome Biol* **19**, 200 (2018).
6. Beh, L.Y. *et al.* Identification of a DNA N6-adenine methyltransferase complex and its impact on chromatin organization. *Cell* **177**, 1781-96 (2019).
7. Fu, Y. *et al.* N6-methyldeoxyadenosine marks active transcription start sites in Chlamydomonas. *Cell* **161**, 879-92 (2015).
8. Liang, Z. *et al.* DNA N(6)-adenine methylation in *Arabidopsis thaliana*. *Dev Cell* **45**, 406-416.e3 (2018).
9. Zhou, C. *et al.* Identification and analysis of adenine N(6)-methylation sites in the rice genome. *Nat Plants* **4**, 554-563 (2018).
10. Zhang, Q. *et al.* N(6)-methyladenine DNA methylation in japonica and indica rice genomes and its association with gene expression, plant development, and stress responses. *Mol Plant* **11**, 1492-1508 (2018).
11. Chen, H. *et al.* Phytophthora methylomes are modulated by 6mA methyltransferases and associated with adaptive genome regions. *Genome Biol* **19**, 181 (2018).
12. Mondo, S.J. *et al.* Widespread adenine N6-methylation of active genes in fungi. *Nat Genet* **49**, 964-8 (2017).
13. Dabe, E.C., Sanford, R.S., Kohn, A.B., Bobkova, Y. & Moroz, L.L. DNA methylation in basal metazoans: Insights from ctenophores. *Integr Comp Biol* **55**, 1096-110 (2015).
14. Greer, E.L. *et al.* DNA methylation on N6-adenine in *C. elegans*. *Cell* **161**, 868-78 (2015).
15. Zhang, G. *et al.* N6-methyladenine DNA modification in Drosophila. *Cell* **161**, 893-906 (2015).
16. Falckenhayn, C. *et al.* Comprehensive DNA methylation analysis of the *Aedes aegypti* genome. *Sci Rep* **6**, 36444 (2016).
17. Wang, X. *et al.* DNA methylation on N6-adenine in lepidopteran *Bombyx mori*. *Biochim Biophys Acta Gene Regul Mech* **1861**, 815-25 (2018).
18. Liu, J. *et al.* Abundant DNA 6mA methylation during early embryogenesis of zebrafish and pig. *Nat Commun* **7**, 13052 (2016).
19. Koziol, M.J. *et al.* Identification of methylated deoxyadenosines in vertebrates reveals diversity in DNA modifications. *Nat Struct Mol Biol* **23**, 24-30 (2016).
20. Wu, T.P. *et al.* DNA methylation on N(6)-adenine in mammalian embryonic stem cells. *Nature* **532**, 329-33 (2016).
21. Yao, B. *et al.* DNA N6-methyladenine is dynamically regulated in the mouse brain following environmental stress. *Nat Commun* **8**, 1122 (2017).
22. Kweon, S.-M. *et al.* An adversarial DNA N6-methyladenine-sensor network preserves Polycomb silencing. *Mol Cell* **74**, 1138-47 (2019).
23. Kigar, S.L. *et al.* N(6)-methyladenine is an epigenetic marker of mammalian early life stress. *Sci Rep* **7**, 18078 (2017).
24. Xiao, C.-L. *et al.* N6-methyladenine DNA modification in the human genome. *Mol Cell* **71**, 306-18 (2018).
25. Xie, Q. *et al.* N6-methyladenine DNA modification in glioblastoma. *Cell* **175**, 1228-1243.e20 (2018).
26. Pacini, C.E., Bradshaw, C.R., Garrett, N.J. & Koziol, M.J. Characteristics and homogeneity of N6-methylation in human genomes. *Sci Rep* **9**, 5185 (2019).
27. Douvlataniotis, K., Bensberg, M., Lentini, A., Gylemo, B. & Nestor, C.E. No evidence for DNA N (6)-methyladenine in mammals. *Sci Adv* **6**, eaay3335 (2020).
28. Flot, J.F. *et al.* Genomic evidence for ameiotic evolution in the bdelloid rotifer *Adineta vaga*. *Nature* **500**, 453-7 (2013).
29. Vakhrusheva, O.A. *et al.* Genomic signatures of recombination in a natural population of the bdelloid rotifer *Adineta vaga*. *Nat Commun* **11**, 6421 (2020).

30. Nowell, R.W. *et al.* Comparative genomics of bdelloid rotifers: Insights from desiccating and nondesiccating species. *PLOS Biology* **16**, e2004830 (2018).
31. Nowell, R.W. *et al.* Evolutionary dynamics of transposable elements in bdelloid rotifers. *eLife* **10**, e63194 (2021).
32. Franch-Gras, L. *et al.* Genomic signatures of local adaptation to the degree of environmental predictability in rotifers. *Sci Rep* **8**, 16051 (2018).
33. Kim, H.S. *et al.* The genome of the freshwater monogonont rotifer *Brachionus calyciflorus*. *Mol Ecol Resour* **18**, 646-55 (2018).
34. Park, J.C. *et al.* The genome of the marine rotifer *Brachionus koreanus* sheds light on the antioxidative defense system in response to 2-ethyl-phenanthrene and piperonyl butoxide. *Aquat Toxicol* **221**, 105443 (2020).
35. Kang, H.M. *et al.* The genome of the marine monogonont rotifer *Brachionus rotundiformis* and insight into species-specific detoxification components in Brachionus spp. *Comp Biochem Physiol Part D Genomics Proteomics* **36**, 100714 (2020).
36. Blommaert, J., Riss, S., Hecox-Lea, B., Mark Welch, D.B. & Stelzer, C.P. Small, but surprisingly repetitive genomes: transposon expansion and not polyploidy has driven a doubling in genome size in a metazoan species complex. *BMC Genomics* **20**, 466 (2019).
37. Favorov, A. *et al.* Exploring massive, genome scale datasets with the GenometriCorr package. *PLoS Comput Biol* **8**, e1002529 (2012).
38. Supek, F., Bošnjak, M., Škunca, N. & Šmuc, T. REVIGO summarizes and visualizes long lists of gene ontology terms. *PLoS One* **6**, e21800 (2011).
39. Li, Z. *et al.* N6-methyladenine in DNA antagonizes SATB1 in early development. *Nature* **583**, 625-630 (2020).
40. Rodriguez, F. & Arkhipova, I.R. Multitasking of the piRNA silencing machinery: Targeting transposable elements and foreign genes in the bdelloid rotifer *Adineta vaga*. *Genetics* **203**, 255-68 (2016).
41. Williams, B.P. & Gehring, M. Stable transgenerational epigenetic inheritance requires a DNA methylation-sensing circuit. *Nat Commun* **8**, 2124 (2017).
42. Greenberg, M.V.C. & Bourc'his, D. The diverse roles of DNA methylation in mammalian development and disease. *Nat Rev Mol Cell Biol* **20**, 590-607 (2019).
43. Jurkowski, T.P. & Jeltsch, A. On the evolutionary origin of eukaryotic DNA methyltransferases and Dnmt2. *PLoS One* **6**, e28104 (2011).
44. Bewick, A.J. *et al.* Diversity of cytosine methylation across the fungal tree of life. *Nat Ecol Evol* **3**, 479-490 (2019).
45. Blow, M.J. *et al.* The epigenomic landscape of prokaryotes. *PLOS Genetics* **12**, e1005854 (2016).
46. Heyn, H. & Esteller, M. An adenine code for DNA: A second life for N6-methyladenine. *Cell* **161**, 710-3 (2015).
47. Luo, G.Z. & He, C. DNA N(6)-methyladenine in metazoans: functional epigenetic mark or bystander? *Nat Struct Mol Biol* **24**, 503-506 (2017).
48. Schiffers, S. *et al.* Quantitative LC–MS provides no evidence for m6dA or m4dC in the genome of mouse embryonic stem cells and tissues. *Angew Chem Int Ed Engl* **56**, 11268-11271 (2017).
49. Lentini, A. *et al.* A reassessment of DNA-immunoprecipitation-based genomic profiling. *Nat Methods* **15**, 499-504 (2018).
50. Edwards, J.R., Yarychkivska, O., Boulard, M. & Bestor, T.H. DNA methylation and DNA methyltransferases. *Epigenetics Chromatin* **10**, 23 (2017).
51. Murphy, J., Mahony, J., Ainsworth, S., Nauta, A. & van Sinderen, D. Bacteriophage orphan DNA methyltransferases: insights from their bacterial origin, function, and occurrence. *Appl Environ Microbiol* **79**, 7547-55 (2013).
52. Padeken, J. *et al.* Synergistic lethality between BRCA1 and H3K9me2 loss reflects satellite derepression. *Genes Dev* **33**, 436-451 (2019).
53. Zhao, X. *et al.* RNAi-dependent Polycomb repression controls transposable elements in Tetrahymena. *Genes Dev* **33**, 348-364 (2019).
54. Frapporti, A. *et al.* The Polycomb protein Ezl1 mediates H3K9 and H3K27 methylation to repress transposable elements in Paramecium. *Nat Commun* **10**, 2710 (2019).
55. Jurkowska, R.Z. *et al.* H3K14ac is linked to methylation of H3K9 by the triple Tudor domain of SETDB1. *Nat Commun* **8**, 2057 (2017).
