# Supplementary material for: Bacterial N4-methylcytosine as an epigenetic mark in eukaryotic DNA
Source: Nat Commun. 2022 Feb 28;13:1072. doi: 10.1038/s41467-022-28471-w (PMC8885841; doi:10.1038/s41467-022-28471-w)
Supplement: Supplementary file 3 — Description of Additional Supplementary Files [file 41467_2022_28471_MOESM3_ESM.docx]

**DESCRIPTION OF ADDITIONAL SUPPLEMENTARY FILES**

File name: **Supplementary Data 1-4** (provided as sheets S1-S4 in a single .XLSX file)

**Supplementary Data 1**. Amino acid sequences of bdelloid N4CMT and Type II subtype β bacterial methyltransferases, with accession numbers from Genbank or REBASE (.fasta format).

**Supplementary Data 2**. Amino acid sequences of SETDB1 proteins from the phylum Rotifera and three representative protostome phyla, with Genbank accession numbers (.fasta format).

**Supplementary Data 3**. MBD- and SET-domain containing proteins in *A. vaga* Av-ref, with Genbank scaffold numbers and protein IDs from Genoscope (.fasta format).

**Supplementary Data 4**. Contigs with residual bacterial sequence joined to AvL1 DNA (.gff format).

File name: **Supplementary Movie** (MP4 file)

**Feeding bdelloid rotifer *Adineta vaga* isolate L1 under polychromatic polarization microscope (video credit: Michael Shribak and Irina Yushenova, Marine Biological Laboratory).**

File name: **Source Data 1**

Description: **Gene ontology analysis of methylated and unmethylated genes** (.XLSX file).

File name: **Source Data 2**

Description: **Source data for bdelloid LINE/Piwi/Ago content and AvMBD protein binding experiments in Fig. 6c-e**  (.XLSX file).

File name: **Reporting Summary**

Description: **Nature Research Reporting Summary Form**
