# Supplementary material for: Bacterial N4-methylcytosine as an epigenetic mark in eukaryotic DNA
Source: Nat Commun. 2022 Feb 28;13:1072. doi: 10.1038/s41467-022-28471-w (PMC8885841; doi:10.1038/s41467-022-28471-w)
Supplement: Supplementary file 6 — Reporting Summary [file 41467_2022_28471_MOESM6_ESM.pdf]

nature research

Corresponding author(s): Irina Arkhipova

Last updated by author(s): January 12, 2022

Reporting Summary

Nature Research wishes to improve the reproducibility of the work that we publish. This form provides structure for consistency and transparency in reporting. For further information on Nature Research policies, see our Editorial Policies and the Editorial Policy Checklist.

Statistics

For all statistical analyses, confirm that the following items are present in the figure legend, table legend, main text, or Methods section.

n/a	Confirmed	
☐	☒	The exact sample size (*n*) for each experimental group/condition, given as a discrete number and unit of measurement
☒	☐	A statement on whether measurements were taken from distinct samples or whether the same sample was measured repeatedly
☐	☒	The statistical test(s) used AND whether they are one- or two-sided<br>*Only common tests should be described solely by name; describe more complex techniques in the Methods section.*
☒	☐	A description of all covariates tested
☒	☐	A description of any assumptions or corrections, such as tests of normality and adjustment for multiple comparisons
☐	☒	A full description of the statistical parameters including central tendency (e.g. means) or other basic estimates (e.g. regression coefficient) AND variation (e.g. standard deviation) or associated estimates of uncertainty (e.g. confidence intervals)
☐	☒	For null hypothesis testing, the test statistic (e.g. *F*, *t*, *r*) with confidence intervals, effect sizes, degrees of freedom and *P* value noted<br>*Give P values as exact values whenever suitable.*
☒	☐	For Bayesian analysis, information on the choice of priors and Markov chain Monte Carlo settings
☒	☐	For hierarchical and complex designs, identification of the appropriate level for tests and full reporting of outcomes
☒	☐	Estimates of effect sizes (e.g. Cohen's *d*, Pearson's *r*), indicating how they were calculated

Our web collection on statistics for biologists contains articles on many of the points above.

Software and code

Policy information about availability of computer code

Data collection	For sequencing data collection and processing, manufacturer's software provided by the Illumina and Pacific Biosciences platforms was used:<br>Casava v 1.8.2. Illumina. https://support.illumina.com/downloads.html<br>bcl2fastq software v2.20 Illumina. https://support.illumina.com/sequencing/sequencing_software/bcl2fastq-conversion-software.html<br>SMRT Analysis Software v2.3.0 Pacific Biosciences. https://www.pacb.com/
Data analysis	add_kaks_to_MCScanX.pl v1.0, R. Nowell, Imperial College. https://zenodo.org/record/891288<br>anvi'o v 2.3.2. Eren, et al. 2015. https://merenlab.org/software/anvio/<br>bedtools v2.27.1 Quinlan and Hall 2010. https://bedtools.readthedocs.io<br>blast+ v2.10.1. Camacho, et al. 2009. https://ftp.ncbi.nlm.nih.gov/blast/executables/blast+/<br>blasr v1.3.1 Chaisson and Tesler 2012. https://github.com/PacificBiosciences/blasr<br>Bowtie v1.1.0 Langmead, et al. 2009. http://bowtie-bio.sourceforge.net<br>Bowtie2 v2.2.5 Langmead and Salzberg 2012. http://bowtie-bio.sourceforge.net/bowtie2/index.shtml<br>BRAKER v2.1.2 Hoff, et al. 2019. https://github.com/Gaius-Augustus/BRAKER<br>Circos v0.69-6 Krzywinski, et al. 2009. http://circos.ca/software/<br>cutadapt v1.9.2 Martin 2011. https://cutadapt.readthedocs.io/en/stable/<br>deepTools2 v3.1.3 Ramirez, et al. 2016. https://deeptools.readthedocs.io<br>FASTX v0.0.13 Toolkit Hannon lab (CSHL). http://hannonlab.cshl.edu/fastx_toolkit/<br>GOseq v1.46.0. Young MD, et al. 2010. https://bioconductor.org/packages/release/bioc/html/goseq.html<br>HmmerWeb version 2.41.2 Eddy 2011. https://www.ebi.ac.uk/Tools/hmmer/search/hmmsearch<br>HOMER Tools v4.11 Heinz, et al. 2010. http://homer.ucsd.edu<br>HTSeq-count v0.6.1 Anders, et al. 2015. https://htseq.readthedocs.io<br>IQTREE v1.6.11 Trifinopoulos, et al. 2016. http://www.iqtree.org/release/v1.6.11<br>ImageQuant TL v.8.1, Cytiva/VWR. https://us.vwr.com/store/product/25990402/imagequant-tl-8-1-cytiva-formerly-ge-healthcare-life-sciences<br>Image Studio™ Lite 5.2.5 software, LI-COR. https://www.licor.com/bio/image-studio-lite/

MACS v1.3 Zhang, et al. 2008. http://liulab.dfci.harvard.edu/MACS/
MACS2 v2.1.0 Zhang, et al. 2008. https://github.com/jsh58/MACS
MCScanX v0.8 Wang, et al. 2012. http://chibba.pgml.uga.edu/mcscan2/
MEME-ChIP v5.4.1 Ma, et al. 2014. http://meme-suite.org/tools/meme-chip
MUSCLE v3.8.31 Edgar, 2004. https://drive5.com/muscle/downloads_v3.htm
PBJelly v15.8.24 English et al. 2012. https://sourceforge.net/projects/pb-jelly/
RepeatMasker v4.0.7 Smit, et al. 2015. http://www.repeatmasker.org/
REVIGO web based. Supek, et al. 2011. http://revigo.irb.hr/
SMRT Analysis Software v2.3.0 PacBio. https://www.pacb.com/
Tophat v2.1.1 Kim, et al. 2013. https://ccb.jhu.edu/software/tophat
Tandem Repeats Finder v4.09 Benson 1999. https://tandem.bu.edu/trf/trf.html

For manuscripts utilizing custom algorithms or software that are central to the research but not yet described in published literature, software must be made available to editors and reviewers. We strongly encourage code deposition in a community repository (e.g. GitHub). See the Nature Research guidelines for submitting code & software for further information.

Data

Policy information about availability of data

All manuscripts must include a data availability statement. This statement should provide the following information, where applicable:

- Accession codes, unique identifiers, or web links for publicly available datasets
- A list of figures that have associated raw data
- A description of any restrictions on data availability

Sequences obtained in this study were deposited under BioProject PRJNA558051 [https://www.ncbi.nlm.nih.gov/bioproject/PRJNA558051] (SRA accession Nos. SRR9886612, SRR9900832-45 for individual SMRT cells).
Avaga_MBL_L1 genome was deposited under accession No. JAGENE000000000 [https://www.ncbi.nlm.nih.gov/nuccore/JAGENE000000000]. The version described in this paper is version JAGENE010000000. Its accession number is GCA_021403095.1 in the NCBI Assembly database [https://www.ncbi.nlm.nih.gov/assembly/GCA_021403095.1].
ChIP-seq, MeDIP-seq and RNA-seq data generated in this study have been deposited in the GEO database under accession No. GSE140049 [https://www.ncbi.nlm.nih.gov/geo/query/acc.cgi?acc=GSE140049], GSE140050[https://www.ncbi.nlm.nih.gov/geo/query/acc.cgi?acc=GSE140050], GSE140051 [https://www.ncbi.nlm.nih.gov/geo/query/acc.cgi?acc=GSE140051] and GSE140052 [https://www.ncbi.nlm.nih.gov/geo/query/acc.cgi?acc=GSE140052].
All materials are freely available to interested researchers upon request, without restrictions.
Custom scripts are available through the GitHub page (https://github.com/cascoamarillo/epigenetics).

Field-specific reporting

Please select the one below that is the best fit for your research. If you are not sure, read the appropriate sections before making your selection.

☒ Life sciences ☐ Behavioural & social sciences ☐ Ecological, evolutionary & environmental sciences

For a reference copy of the document with all sections, see nature.com/documents/nr-reporting-summary-flat.pdf

Life sciences study design

All studies must disclose on these points even when the disclosure is negative.

Sample size	For comparison between rotifer species, we used all sequenced isolates available in GenBank for each species. For 4 strains, two assemblies obtained using different methods were treated as technical replicates; otherwise, each strain was considered a biological replicate. For quantitative analysis of DNA-protein binding efficiency in competition experiments, gel mobility shift assays were performed five times using a range of concentrations indicated in the text; all experiments showed higher competition efficiency for 4mC-methylated DNA.
Data exclusions	No data were excluded, everything is available in the Source Data files.
Replication	Immuno-dot-blots were replicated five times using several substrate concentrations with 6mA serving as an internal control and with positive and negative substrate controls for each experiment, with all experiments presented in a source data file. Methylated DNA loci showed good agreement when using orthogonal methods such as MeDIP-seq and SMRT-seq. In competition experiments with the methyl-binding domain variant, all attempts at replication were successful.
Randomization	No comparisons were made between experimental groups
Blinding	No group allocations were performed and therefore blinding was not relevant.

Reporting for specific materials, systems and methods

We require information from authors about some types of materials, experimental systems and methods used in many studies. Here, indicate whether each material, system or method listed is relevant to your study. If you are not sure if a list item applies to your research, read the appropriate section before selecting a response.

Materials & experimental systems

n/a	Involved in the study
☐	☒ Antibodies
☒	☐ Eukaryotic cell lines
☒	☐ Palaeontology and archaeology
☐	☒ Animals and other organisms
☒	☐ Human research participants
☒	☐ Clinical data
☒	☐ Dual use research of concern

Methods

n/a	Involved in the study
☐	☒ ChIP-seq
☒	☐ Flow cytometry
☒	☐ MRI-based neuroimaging

Antibodies

Antibodies used

Rabbit polyclonal ChIP-seq grade affinity-purified anti-H3K4me3 Diagenode C15410003 https://www.diagenode.com/en/p/h3k4me3-polyclonal-antibody-premium-sample-size-10-ug Lot No. A1052D
Rabbit polyclonal ChIP-seq grade affinity-purified anti-H3K9me3 Diagenode C15410056 https://www.diagenode.com/en/p/h3k9me3-polyclonal-antibody-classic-sample-size-10-ug Lot No. A1675-001P
Rabbit polyclonal ChIP-seq grade affinity-purified anti-H3K27me3 Diagenode C15410195 https://www.diagenode.com/en/p/h3k27me3-polyclonal-antibody-premium-sample-size-10-ug Lot No. A1811-001P
Rabbit primary anti-4mC from Dr. Ian Murray (NEB): Kong, H. et al. Functional analysis of putative restriction-modification system genes in the Helicobacter pylori J99 genome. Nucl Acids Res 28, 3216-23 (2000).
Rabbit primary anti-4mA from Dr. Ian Murray (NEB): Kong, H. et al. Functional analysis of putative restriction-modification system genes in the Helicobacter pylori J99 genome. Nucl Acids Res 28, 3216-23 (2000).
Monoclonal His Tag Antibody. Aviva Systems Biology. Cat # OAEA00010. Clone HIS.H8. Isotype IgG2b. Lot #01014. https://www.avivasysbio.com/his-tag-antibody-oaea00010.html
S•Tag™ Monoclonal Antibody. Sigma-Aldrich. Cat # 71549-3. Mouse Isotype IgG2b. Lot #D00145366. https://www.emdmillipore.com/US/en/product/STag-Monoclonal-Antibody,EMD_BIO-71549
Goat anti-mouse IgG-HRP. Santa Cruz Biotechnology. Cat # sc-2005. Lot #G3114. https://www.scbt.com/p/goat-anti-mouse-igg-hrp
Goat anti-rabbit-HRP antibody, MilliporeSigma, Cat. # A0545. https://www.sigmaaldrich.com/US/en/product/sigma/a0545

Validation

Premium ChIP-seq grade affinity-purified rabbit polyclonal antibodies, raised against synthetic peptides with the corresponding trimethylated lysines, according to the manufacturer display a wide range of species reactivity including vertebrates, Drosophila, C. elegans and plants, and have been validated by ChIP-seq, IF, Western blotting, and ELISA (as described on the Diagenode websites above). The H3 N-terminal residues 1-31 display 100% identity between A. vaga and humans, and no cross-reactivity of K9/K27 was observed in human peptide arrays spanning the identical aa sequence (Diagenode). Each antibody was tested in our laboratory by Western blotting prior to use in ChIP-seq experiments. Anti-4mC and anti-4mA antibodies were assayed for specificity by immuno-dot-blotting using the control unmethylated, 4mC-methylated and 6mA-methylated bacterial DNA, as well as 5mC-methylated human DNA. Before Western blotting of recombinant proteins, optimal working dilution for His tag, S•Tag and anti-mouse IgG-HRP antibodies were determined by titration test, according to manufacturers' guidelines.

Animals and other organisms

Policy information about studies involving animals; ARRIVE guidelines recommended for reporting animal research

Laboratory animals

Freshwater invertebrates: Adineta vaga (Rotifera; Bdelloidea), strains Av-ref (established from single egg in 1995) and AvL1 (established from single egg in 2013), maintained as laboratory cultures, all-female

Wild animals

The study did not involve wild animals

Field-collected samples

The study did not involve samples collected from the field

Ethics oversight

No ethical approval or guidance is required for microscopic invertebrate animals

Note that full information on the approval of the study protocol must also be provided in the manuscript.

ChIP-seq

Data deposition

☒ Confirm that both raw and final processed data have been deposited in a public database such as GEO.

☒ Confirm that you have deposited or provided access to graph files (e.g. BED files) for the called peaks.

Data access links
May remain private before publication.

https://www.ncbi.nlm.nih.gov/geo/query/acc.cgi?acc=GSE140052

Files in database submission

Av11-H3K4_peaks.bed, Av11-H3K9_peaks.bed, Av11-H3K27_peaks.bed, Av-H3K4_peaks.bed, Av-H3K9_peaks.bed, Av-H3K27_peaks.bed, Av11-H3K4_S5_R1_001.fastq.gz, Av11-H3K9_S6_R1_001.fastq.gz, Av11-H3K27_S7_R1_001.fastq.gz,

	Av11-Input_S8_R1_001.fastq.gz, Av-H3K4_S1_R1_001.fastq.gz, Av-H3K9_S2_R1_001.fastq.gz, Av-H3K27_S3_R1_001.fastq.gz, Av-Input_S4_R1_001.fastq.gz
Genome browser session (e.g. UCSC)	N/A

Methodology

Replicates	For each strain, immunopercipitated DNA was sequenced along with DNA input control; histone methylation in two strains showed highly similar patterns.
Sequencing depth	total reads (Million) per experiment: Av11-H3K4_S5_R1_001.fastq (16.8M), Av11-H3K9_S6_R1_001.fastq (12.9M), Av11-H3K27_S7_R1_001.fastq (15.5M), Av11-Input_S8_R1_001.fastq (24.3M), Av-H3K4_S1_R1_001.fastq (23.1M), Av-H3K9_S2_R1_001.fastq (12.7M), Av-H3K27_S3_R1_001.fastq (19.5M), Av-Input_S4_R1_001.fastq 16.5M. Sequenced on Illimuna NextSeq 500 for 75 bp single-end High-Throughput
Antibodies	Rabbit anti-H3K4me3 Diagenode Cat. #15410003, Rabbit anti-H3K9me3 Diagenode Cat. #15410056, Rabbit anti-H3K27me3 Diagenode Cat. #15410195, 1 µg each
Peak calling parameters	MACS2 was used with default settings for peak calling
Data quality	21067 peaks were at 1E-2 p-value cutoff and above 5 fold enrichment
Software	MACS2 (Zhang et al., 2008)
